# Practical Considerations When Using Mendelian Sampling Variances for Selection Decisions in Genomic Selection Programs

**DOI:** 10.1111/jbg.12913

**Published:** 2024-12-02

**Authors:** Tobias A. M. Niehoff, Jan ten Napel, Mario P. L. Calus

**Affiliations:** ^1^ Animal Breeding and Genomics Wageningen University & Research Wageningen the Netherlands

**Keywords:** breeding planning, genetic variance, Mendelian sampling variance, progeny variance, selection decisions, usefulness criterion

## Abstract

The purpose of this work was to test the application of selection criteria that consider the genetic variances of future generations. This has not been done previously in numerically large livestock breeding programs based on estimated rather than assumed known marker effects. A generic pure‐line pig breeding program was simulated in which 40 males and 400 females were selected every generation. Daily gain was used as an example trait. Three variance‐considering criteria as well as different reference population sizes were compared to investigate the effect of differences in genomic prediction accuracy on selection decisions. All variance‐considering criteria retained more genetic variance than if selection was based on estimated breeding values (max. 20%). This effect was more pronounced for higher prediction accuracies and criteria assessing the variance more generations ahead. After 20 generations, the criterion with the longest planning horizon combined with the largest reference population resulted in a 2% higher genetic level of boars selected to produce finisher pigs. While the advantage of accounting for future generations diminished with lower accuracy or shorter planning horizons, the variance‐considering criteria never performed worse than selection based on genomic estimated breeding values (GEBV) with respect to commercial genetic gain. We are reporting various accuracy metrics to help judge the effectiveness of using one of our tested criteria in real breeding programs. While we did not find large benefits for genetic gain when considering future variances in selection decisions, we also did not find negative side effects, while considerably more genetic variance was maintained. This means that using variance‐considering criteria results in either equally good or better performance than truncation selection based on estimated breeding values. Our criteria can be applied to any genomic breeding program as long as phased genotypes, estimated marker effects and a genetic map are available.

## 1 Introduction

Genetic gain in commercial breeding programs is generated by selecting the best animals based on estimated breeding values as parents of the next generation. Selecting the best‐performing animals as parents maximises the average performance of the next generation. If only a fraction of the offspring generation is of interest, typically the highest‐ranking offspring whose genetic material will be disseminated, the progeny variance should also be taken into account in addition to their breeding values when selecting parents. For this, several selection criteria have been proposed (Bijma, Wientjes, and Calus 2020; Müller, Schopp, and Melchinger 2018; Santos et al. 2019; Schnell and Utz 1975; Shepherd and Kinghorn 1998; Wellmann and Bennewitz 2019). Long‐term selection simulation studies that test these criteria in realistic breeding programs with estimated effects have been published in plant breeding research (Allier et al. 2019; Michel et al. 2022; Neyhart, Lorenz, and Smith 2019; Sanchez et al. 2023). Investigations for animal breeding programs focus solely on the next generation (Bijma, Wientjes, and Calus 2020), simulate a low number of quantitative trait loci (QTL) (Santos et al. 2019) or assume that QTL effects and thus progeny variances are known without error (Bijma, Wientjes, and Calus 2020; Musa and Reinsch 2023; Niehoff et al. 2024), but research based on estimated effects is scarce.

Recently, we developed analytical equations to predict the progeny variance in any desired future generation, which allows for developing selection criteria that maximise the performance of the best animals in the grand‐offspring, great‐grand‐offspring, etc., generation (Niehoff, ten Napel, and Calus 2024). Although the gametic Mendelian sampling variance (MSV) of individuals can be estimated with medium to high accuracy as reported for dairy cattle (Hozé et al. 2022; Linders 2020; Santos et al. 2019; Segelke et al. 2014), no work has examined the effect that this imperfect estimation has on the breeding program in the long term. This was the purpose of this study. In addition, we test our newly developed selection criteria (Niehoff et al. 2024; Niehoff, ten Napel, and Calus 2024) and Index5 (Bijma, Wientjes, and Calus 2020) in a simulation study of a simplified pig breeding scheme. We test the effect of (i) the number of generations a criterion is planning ahead and (ii) the influence of the genomic prediction training population size. We investigate the impacts on (a) genetic gain, (b) genetic diversity and (c) the accuracy of genomic prediction.

## 2 Materials and Methods

In this study, we simulate a generic pig breeding scheme to investigate the use of MSV‐considering criteria. We examine the outcomes for different training population sizes and selection criteria and report several parameters to judge the effectiveness of including the MSV in selection decisions.

### 2.1 Pig Breeding Program Simulation

A computer simulation study of a generic pig breeding program was conducted to investigate the benefit of using MSV‐based selection criteria. The simulation software MoBPS version 1.10.68 was used (Pook, Schlather, and Simianer 2020). Every scenario was tested with 100 replicates. The code for this study was run in R version 4.1.2 on the high‐performance cluster Anunna of Wageningen University & Research.

We simulated 18 chromosome pairs, each of which had a length of 1 Morgan. To obtain base population genomes for an effective population size of Ne = 100, we modelled a historical population of 4×Ne=400 generations of random mating with a population size of 100 in every generation, following Meuwissen et al. (2020). We simulated 1,000,000 base pairs (loci) and used a mutation rate of 5 × 10^−5^ during those 400 generations. All loci were fully fixed for the 0 allele at the start. Approximately 64,000 to 66,000 loci were segregating after 400 generations. The observed heterozygosity, $H_{o}$, matched the expectation according to $H_{e}=4N_{e}u{\left(4N_{e}u+1\right)}^{-1}$ based on Kimura and Crow (1964), with effective population size $N_{e}$ and mutation rate u. For computational reasons, we removed all loci with minor allele frequencies lower than 0.02 from the genome, i.e., loci at which only three copies or fewer were observed for the minor allele in the population. Meiosis was simulated with default parameters in MoBPS (Pook, Schlather, and Simianer 2020).

We then assigned additive effects to 6000 randomly chosen segregating loci. The effects were drawn from a gamma distribution with a shape parameter of 0.4, which results in many QTLs with small effect alleles and a few with larger effects. We then chose 20,000 loci to be included in the genotyping array. Only loci without effects on the trait were considered for the array. Loci for the single‐nucleotide polymorphism (SNP) array were chosen as follows: the genome was divided into 20,000 equally sized bins. Within each bin, the locus with the highest minor allele frequency (MAF) was selected for the genotyping array. This procedure allows for covering the genome evenly and for selecting SNPs in such a way that they allow maximum differentiation between individuals. As a consequence, the MAF distribution of all SNPs was L‐shaped, whereas the MAF distribution of SNPs on the genotyping array was approximately uniform (see Figure SF1), which is generally the case for commercial SNP chips (Eynard et al. 2015; Groenen et al. 2011; Matukumalli et al. 2009; Ramos et al. 2009).

Following the population history simulation, 10 generations of recurrent genomic selection were simulated as a burn‐in period to reach Bulmer equilibrium. Every generation, 40 males and 400 females were selected out of 1200 male and 1200 female selection candidates as parents for the next generation based on their genomic estimated breeding value, as in Wientjes, Bijma, and Calus (2020). Males and females had equal contributions within sex. Each male was mated to 10 females. The females were fully nested within males, i.e., each female had one litter and all six piglets of a litter were sired by the same male, as in Wientjes, Bijma, and Calus (2020).

After the initial 10 generations, the breeding program was run for another 19 generations. The number of selection candidates and selected sires and dams as well as litter sizes in the breeding program were the same as during the burn‐in period. Selection was based on one of five different criteria, as described in the next subsection. The 40 best males and 400 females according to the respective selection criterion were selected as parents of the next generation and mated randomly. Of all the males that were not selected as sires of the next generation, the top 80 were selected based on their GEBV as artificial insemination (AI) boars to serve the market. These boars could be used to produce crossbred finisher animals on production farms or to distribute genetic progress from the nucleus population to multiplier farms, as in Wientjes, Bijma, and Calus (2020).

We modelled a trait with a moderate heritability of 0.3 for which all animals were phenotyped. This may resemble a simple trait like daily gain in pigs (Gao et al. 2021; Kavlak and Uimari 2019). All animals were genotyped before breeding values were estimated and selection decisions were made. Genomic breeding value estimation was performed within MoBPS. The relationship matrix was created based on method 1 of VanRaden (2008) using those allele frequencies that were observed in the first generation of the burn‐in period, considering this to be the base population. SNP effects were calculated in MoBPS by backsolving.

To investigate the effect of different prediction accuracies, we used three different training population sizes. The training populations were (A) only animals of the current generation, (B) the current generation + the past two generations and (C) the current generation + the past five generations.

### 2.2 Selection Criteria

We used five different strategies for selection in this study: random selection, GEBV, Index5 (Bijma, Wientjes, and Calus 2020), ExpBVSelGrOff and ExpBVSelGrGrOff (Niehoff et al. 2024; Niehoff, ten Napel, and Calus 2024). Starting with the Index5, each criterion aims at maximising the population average breeding value one generation further ahead than the previous criterion. Table 1 lists definitions of the parameters utilised for the calculation of the selection criteria.

**TABLE 1:** Definition of parameters to calculate selection criteria.

${\mathrm{BV}}_{\text{sire}}$	Breeding value of a sire
${\mathrm{BV}}_{\mathrm{dam}}$	Breeding value of a dam
${\mathrm{BV}}_{\text{animal}}$	Breeding value of an animal
$\sigma_{{\text{gamMS}}_{\text{sire}}}^{2}$	Gametic Mendelian sampling variance of a sire
$\sigma_{{\text{gamMS}}_{\mathrm{dam}}}^{2}$	Gametic Mendelian sampling variance of a dam
$\sigma_{{\text{gamMS}}_{\text{animal}}}^{2}$	Gametic Mendelian sampling variance of an animal
$\sigma_{\mathrm{FS}}^{2}$	Variance of breeding values of full‐sib offspring of a mating $\sigma_{{\text{gamMS}}_{\text{sire}}}^{2}+\sigma_{{\text{gamMS}}_{\mathrm{dam}}}^{2}$
${\bar{\sigma }}_{{\text{gamMS}}_{\mathrm{off}}}^{2}$	Average gametic Mendelian sampling variance of offspring of a mating
${\mathrm{BV}}_{A};{\mathrm{BV}}_{B};{\mathrm{BV}}_{C};{\mathrm{BV}}_{D}$	Breeding values of animals A, B, C and D
$\sigma_{{\text{gamMS}}_{A}}^{2};\sigma_{{\text{gamMS}}_{B}}^{2};\sigma_{{\text{gamMS}}_{C}}^{2};\sigma_{{\text{gamMS}}_{D}}^{2}$	Gametic MSV of animals A, B, C and D
$\sigma_{{\mathrm{FS}}_{\mathrm{AxB}}}^{2};\sigma_{{\mathrm{FS}}_{\mathrm{CxD}}}^{2}$	Variance of BV among full‐sib offspring of the mating between animal A with B and C with D $\sigma_{{\text{gamMS}}_{A}}^{2}+\sigma_{{\text{gamMS}}_{B}}^{2};\sigma_{{\text{gamMS}}_{C}}^{2}+\sigma_{{\text{gamMS}}_{D}}^{2}$
${\bar{\sigma }}_{{\text{gamMS}}_{\mathrm{off}\;\mathrm{AxB}}}^{2};{\bar{\sigma }}_{{\text{gamMS}}_{\mathrm{off}\;\mathrm{CxD}}}^{2}$	Average gametic MSV of offspring of the mating A × B and C × D
$\sigma_{{\mathrm{off}}_{\left(\mathrm{AxB}\right)x\left(\mathrm{CxD}\right)}}^{2}$	Variance of breeding values of offspring of the matings between selected offspring of A × B and selected offspring of C × D $\frac{\left(1-k_{i}\right)\sigma_{{\mathrm{FS}}_{\mathrm{AxB}}}^{2}}{4}+\frac{\left(1-k_{i}\right)\sigma_{{\mathrm{FS}}_{\mathrm{CxD}}}^{2}}{4}+{\bar{\sigma }}_{{\text{gamMS}}_{\mathrm{off}\;\mathrm{AxB}}}^{2}+{\bar{\sigma }}_{{\text{gamMS}}_{\mathrm{off}\;\mathrm{CxD}}}^{2}$
${\bar{\sigma }}_{{\text{gamMS}}_{\mathrm{off}\;\left(\mathrm{AxB}\right)x\left(\mathrm{CxD}\right)}}^{2}$	Average gametic MSV of offspring of the matings between selected offspring of AxB and selected offspring of C × D
$i_{i}$	Selection intensity belonging to the selected proportion of the population for population improvement; in this study, $i_{i}=1.6589$
$x_{i}$	Normalised truncation selection point belonging to the selected proportion of the population for population improvement; in this study, $x_{i}=1.1323$
$k_{i}$	Variance reduction coefficient belonging to the selected proportion of the population for population improvement; in this study, $k_{i}=0.7977$
$i_{m}$	Selection intensity belonging to the selected proportion of AI boars for genetic dissemination to production farms (market); in this study, $i_{m}=1.9396$
$x_{m}$	Normalised truncation selection point belonging to the selected proportion of AI boars for genetic dissemination to production farms (market); in this study, $x_{m}=1.501$

The Index5 corresponds to the linearized probability of an individual to produce top‐ranking offspring. The Index5 was calculated with Equation (1) according to Bijma, Wientjes, and Calus (2020).

(1) $$I_{5}={\mathrm{BV}}_{\text{animal}}+x_{m}\times \sqrt{{2\times \sigma }_{{\text{gamMS}}_{\text{animal}}}^{2}}$$

The ExpBVSelGrOff criterion aims at predicting the expected breeding value of selected grand‐offspring. We modified the ExpBVSelGrOff criterion presented in Niehoff et al. (2024) so that different selection intensities for population improvement and marketable sires are accounted for. In addition, we removed all population‐dependent components. These are instead approximated by the respective counterpart components of the mating (Equation 2) following the style of the selection criterion presented in Niehoff, ten Napel, and Calus (2024). All future variance components were predicted as explained in Niehoff et al. (2024) and Niehoff, ten Napel, and Calus (2024).

(2) $$\begin{array}{cc} \text{ExpBVSelGrOff}\;=\; & \frac{{\mathrm{BV}}_{\text{sire}}+{\mathrm{BV}}_{\mathrm{dam}}}{2}+i_{i}\sqrt{\sigma_{\mathrm{FS}}^{2}}\\ & +\sqrt{2}i_{m}\sqrt{\frac{\left(1-k_{i}\right)\sigma_{\mathrm{FS}}^{2}}{4}+{\bar{\sigma }}_{{\text{gamMS}}_{\mathrm{off}}}^{2}} \end{array}$$

With $k_{i}=i_{i}\times \left(i_{i}-x_{i}\right)$.

The ExpBVSelGrGrOff criterion was calculated as shown in (3) following the style of Niehoff, ten Napel, and Calus (2024).

(3) $$\begin{array}{c} \text{ExpBVSelGrGrOff}\\ =\frac{{\mathrm{BV}}_{A}+{\mathrm{BV}}_{B}+{\mathrm{BV}}_{C}+{\mathrm{BV}}_{D}}{4}+i_{i}\left(\frac{\sqrt{\sigma_{{\mathrm{FS}}_{\mathrm{AxB}}}^{2}}+\sqrt{\sigma_{{\mathrm{FS}}_{\mathrm{CxD}}}^{2}}}{2}\right)\\ +\;i_{i}\sqrt{\frac{\left(1-k_{i}\right)\sigma_{{\mathrm{FS}}_{\mathrm{AxB}}}^{2}}{4}+\frac{\left(1-k_{i}\right)\sigma_{{\mathrm{FS}}_{\mathrm{CxD}}}^{2}}{4}+{\bar{\sigma }}_{{\text{gamMS}}_{1\;\mathrm{off}\;\mathrm{AxB}}}^{2}+{\bar{\sigma }}_{{\text{gamMS}}_{\mathrm{off}\;\mathrm{CxD}}}^{2}}\\ +\;i_{m}\sqrt{2}\sqrt{\frac{\left(1-k_{i}\right)\sigma_{{\mathrm{off}}_{\left(\mathrm{AxB}\right)x\left(\mathrm{CxD}\right)}}^{2}}{4}+{\bar{\sigma }}_{{\text{gamMS}}_{\mathrm{off}\;\left(\mathrm{AxB}\right)x\left(\mathrm{CxD}\right)}}^{2}} \end{array}$$

With $k_{i}=i_{i}\times \left(i_{i}-x_{i}\right)$.

The selection intensity ($i_{i}$), normalised truncation selection point ($x_{i}$) and variance reduction coefficient ($k_{i}$) for the population improvement part are the average of the values of the male and female selection path. The selection intensity and normalised truncation selection point belonging to AI boars to serve market needs ($i_{m},x_{m}$) are based on the selected fraction of AI boars for production (80/1200). Note that we chose the values for $i_{m}$ and $x_{m}$ to correspond to the best case, i.e., the AI boars are boars 1 to 80 according to their GEBVs. In the worst case, these are boars 41–120, which would strictly require different values for $i_{m}$ and $x_{m}$. The reality would be somewhere between these two extremes. For simplicity, we assumed the value for the best case.

### 2.3 Selection Algorithm

Since the ExpBVSelGrOff and ExpBVSelGrGrOff criteria, respectively, express values for a pair and a quartet of animals, selection is less straightforward than simply sorting a list of animals based on their GEBVs or Index5 values. Thus, we designed an algorithm to select the parents of the next generation when using ExpBVSelGrOff and ExpBVSelGrGrOff. In addition, evaluating all possible matings for the ExpBVSelGrOff criterion is computationally demanding, since the gametic MSV of offspring for all 1200 × 1200 = 1,440,000 possible matings need to be calculated. This problem increases for the ExpBVSelGrGrOff criterion as (1200 × 1200)^2^ = 2,073,600,000,000 quartets are possible and require the calculation of the gametic MSV of grand‐offspring. To address the two issues of (1) how to select individuals when information is expressed for pairs or quartets and (2) how to find a solution when searching the entire solution space is computationally too demanding, we utilised a selection approach inspired by how one might select based on general combining ability (GCA) in hybrid breeding (Supporting Information SM1). In brief, the number of selection candidates was iteratively reduced, based on the highest average value of every selection candidate for ExpBVSelGrOff or ExpBVSelGrGrOff. These average values were computed considering a limited number of mates, called ‘testers’ in hybrid breeding, for each selected animal. The number of mates (testers) considered to compute those averages grew progressively across iterations as the number of retained selection candidates decreased. This strategy ultimately aims to select animals with the highest average ExpBVSelGrOff and ExpBVSelGrGrOff values (analogous to GCAs).

### 2.4 Analysed Parameters

We analysed several metrics to assess how well the different criteria performed. These can be grouped into metrics related to genetic gain, genetic diversity and the accuracy and similarity of selection decisions. We investigated:

For gain:

1. Conventional genetic level as the population average true breeding value
2. Commercial genetic level as the average true breeding value of the 80 AI boars selected to disseminate genetic progress to production farms

For diversity:

3 Average inbreeding level based on identity by descent (IBD) with the first burn‐in generation as the unrelated founder generation, as in Niehoff et al. (2024)
4 Number of beneficial alleles that were lost
5 True genic variance as the sum over all QTLs of $2{\mathrm{pq\alpha}}^{2}$
6 True genetic variance as variance of true breeding values of selection candidates
7 True genetic variance corrected for the Bulmer effect as four times the average true gametic MSV
8 True genetic variance after selection pressure is released as the variance over the true breeding values of animals obtained after five generations of random mating without selection, as in Niehoff et al. (2024)

The difference among approaches 5–8 is that the genic variance does not account for linkage disequilibrium, and thus covariance, between loci whereas the last three metrics do. With directional selection, the Bulmer effect causes a negative covariance between beneficial alleles, consequently making the breeding values of the group of selected individuals appear more alike relative to a group of randomly chosen individuals (Falconer and Mackay 1996, p. 202). In simple terms, the higher the selection intensity, the smaller the variance of possible parent average breeding values, and thus the lesser the variance of the next generation. Since considering the gametic MSV or the MSV of descendants in addition to the breeding value decreases the emphasis on the breeding values, and accordingly the selection differential (difference between population average and average of selected parents), the variance of parent average breeding values is reduced less. Therefore, by design, any criterion that considers a property additional to the breeding value will result in seemingly higher genetic variance. To eliminate the bias in favour of MSV‐considering criteria, we use five generations of random mating without selection at a population size of 1000 in metric 8.

The gametic phase disequilibrium is halved with every generation of random mating without selection for unlinked loci (Falconer and Mackay 1996, p. 202). Thus, after five generations, 96.875% (1–0.5^5^) of the difference of the genetic variance of the current generation to the variance of the unselected base population is restored if the average kinship in the population does not increase. Therefore, the variance metric 8 and the genic variance (metric 5) should yield approximately similar values. However, genes are linked to chromosomes. Using several generations of random mating to reduce the Bulmer effect also decreases any beneficial or detrimental gametic phase disequilibrium. The average within‐family variance is half the total genetic variance in the (hypothetical) parental generation that is not inbred, was created by random mating and is unaffected by selection. The within‐family variance is the sum of the gametic MSV of both parents (Walsh and Lynch 2018, chapter 16). Consequently, the average gametic MSV is a quarter of the total additive genetic variance of populations not affected by selection. We utilised four times the average gametic MSV for our metric 7 to account for differences in the reduction of parent average variances while keeping the same within‐chromosome linkage disequilibrium structure as observed in the population. The multiplication by 4 brings the variances of metrics 5, 6, 7 and 8 to a comparable level.

Accuracy and similarity of selection decisions:

9 Accuracy of GEBVs for all animals in the training population as the correlation between true and estimated breeding values
10 Accuracy of GEBVs for all selection candidates (current generation) as the correlation between true and estimated breeding values
11 Accuracy of estimated Mendelian sampling terms as the average correlation between true and estimated breeding values of the six members of each of the 400 full‐sib families
12 Correlation between true and estimated gametic MSV of selection candidates
13 The ratio of the average estimated gametic MSV relative to the average estimated variance within a full‐sib family
14 The fraction of males and females selected that would have otherwise also been selected based on their GEBVs
15 Correlation between estimated gametic MSV and inbreeding levels of selection candidates

The accuracy of GEBVs for all animals in the training population (metric 9) represents that of the genomic prediction model. Metric 10, which only focuses on the GEBV accuracy for selection candidates, corresponds to that of the model for making selection decisions. The average correlation between true breeding values (TBV) and EBVs per full‐sib family in metric 11 indicates the accuracy of the estimated Mendelian sampling terms and corrects for differences in parent average breeding values between the full‐sib families. Thus, this metric describes how well the model predicts Mendelian sampling terms. In other words, this removes the predictive power that the parent average breeding value has on the breeding value of the candidates.

For metric 12, we calculated the correlation between the true and the estimated gametic MSV of selection candidates.

To assess whether the shrinkage acting on the gametic MSV is different from that acting on the GEBVs of the selection candidates, we calculated the ratio of the average gametic MSV relative to the average full‐sib variance $\left(\frac{{\bar{\sigma }}_{\text{gamMS}}^{2}}{{\bar{\sigma }}_{\mathrm{FS}}^{2}}\right)$ (metric 13). If the shrinkage is the same for estimated gametic MSV as it is for GEBVs, then the ratio is expected to be 0.5, as the average gametic MSV for non‐inbred individuals is half the additive genetic variance within full‐sib families (Dempfle 1990). If the ratio is smaller (larger) than 0.5, then this would indicate more (less) shrinkage of the gametic MSV relative to the breeding values, and thus less (more) emphasis on the gametic MSV compared to the breeding values in the MSV‐considering selection criteria. We used the average estimated gametic MSV of the selection candidates, their GEBVs and the GEBVs of their parents to calculate this ratio. We corrected for the inbreeding level since differences in the inbreeding level of candidates compared to their parents can result in ratios differing from 0.5 even without differences in shrinkage.

The ratio was calculated as:

$$\frac{\frac{\sum_{i=1}^{N}\sigma_{{\text{gamMS}}_{i}}^{2}*\frac{1}{\left(1-F_{i}\right)}}{N}}{\frac{\sum_{i=1}^{N}{\left(\left({\mathrm{BV}}_{i}-0.5\left({\mathrm{BV}}_{{\text{sire}}_{i}}+{\mathrm{BV}}_{{\mathrm{dam}}_{i}}\right)\right)\times \sqrt{\frac{1}{\left(1-\frac{F_{{\text{sire}}_{i}}+F_{{\mathrm{dam}}_{i}}}{2}\right)}}\right)}^{2}}{N}}$$

Which can be rewritten as:

$$\frac{\sum_{i=1}^{N}\sigma_{{\text{gamMS}}_{i}}^{2}{\left(1-F_{i}\right)}^{-1}}{\sum_{i=1}^{N}{\left({\mathrm{BV}}_{i}-0.5\left({\mathrm{BV}}_{{\text{sire}}_{i}}+{\mathrm{BV}}_{{\mathrm{dam}}_{i}}\right)\right)}^{2}{\left(1-0.5\left(F_{{\text{sire}}_{i}}+F_{{\mathrm{dam}}_{i}}\right)\right)}^{-1}}$$

where N is the number of all selection candidates, $\sigma_{{\text{gamMS}}_{i}}^{2}$ is the estimated gametic MSV of individual i, $F_{i}$ is the inbreeding level of individual i, ${\mathrm{BV}}_{i}$ is the GEBV of individual i and ${\mathrm{BV}}_{{\text{sire}}_{i}}$, ${\mathrm{BV}}_{{\mathrm{dam}}_{i}}$, $F_{{\text{sire}}_{i}}$ and $F_{{\mathrm{dam}}_{i}}$ are the GEBVs and inbreeding levels of the sire and dam of individual i, respectively. We subtracted 1 off of the diagonal elements of the genomic relationship matrix based on VanRaden (2008) method 1 to obtain the inbreeding level. This matrix was computed based on the 20,000 SNPs on the genotyping array, using the allele frequencies of the first generation of the burn‐in period.

For metric 14, we checked what fraction of males (females) selected based on one of the selection criteria are among the top 40 (400) males (females) if ranked according to their GEBVs.

For metric 15, the inbreeding level derived from the genomic relationship matrix was correlated to the estimated gametic MSV of all selection candidates.

## 3 Results

### 3.1 Genetic Progress

**FIGURE 1:**
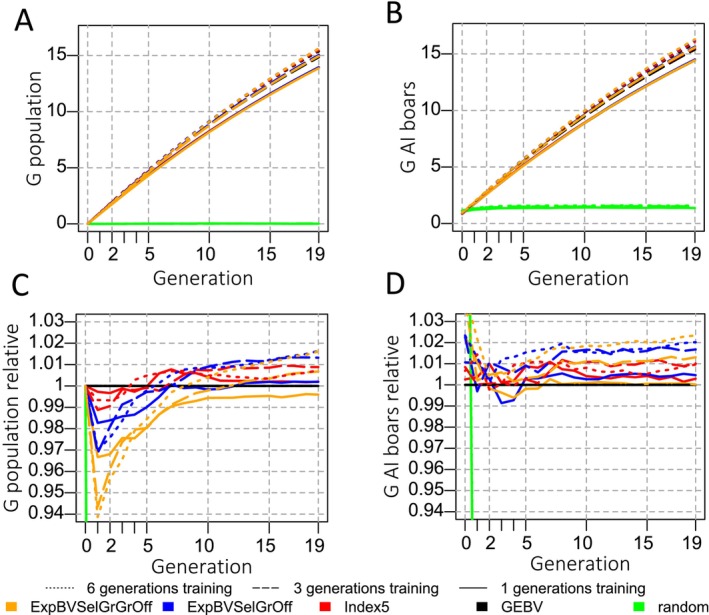
Evolution of genetic levels for different groups of individuals: (A) the average true breeding value of the population (metric 1), (B) the average true breeding value of 80 males selected as AI boars for dissemination of genetic progress to production animals expressed as the difference to the population average breeding value in generation 0 in units of genetic standard deviation (metric 2). Plots C and D show trajectories expressed relative to selection based on GEBVs. The expression of the genetic level in plots C and D needs to be understood as a ratio, e.g., 1.01 means that the genetic level was 1.01 times, or 1%, larger than the genetic level in the GEBV selection scenario. Ratios were calculated in this way: $\frac{{\bar{\mathrm{BV}}}_{\mathrm{pop}\;{\text{crit}}_{{\mathrm{gen}}_{t}}}-{\bar{\mathrm{BV}}}_{\mathrm{pop}\;{\text{GEBV}}_{{\mathrm{gen}}_{0}}}}{{\bar{\mathrm{BV}}}_{\mathrm{pop}\;{\text{GEBV}}_{{\mathrm{gen}}_{t}}}-{\bar{\mathrm{BV}}}_{\mathrm{pop}\;{\text{GEBV}}_{{\mathrm{gen}}_{0}}}}$. The standard errors of the mean difference of the ratios were very similar among the tested scenarios (excluding random selection). For simplicity, we only report the highest standard error, which was 0.0038. [Colour figure can be viewed at wileyonlinelibrary.com]

The highest genetic progress was realised with the largest training population (Figure 1A,B). Within the same training population size, the genetic progress measured as the average breeding value of the population was generally highest when selecting based on breeding values up to generations 5–10 (see Figure 1C). Based on a paired t‐test, all MSV‐considering criteria performed significantly (alpha = 0.05, two‐sided) worse in early generations than if selection was based on breeding values. In the last generation, the genetic level achieved with MSV‐considering criteria differed significantly from the level achieved with GEBV‐based selection when using 3 or 6 generations in the training population. If the training population only consisted of the current generation animals, genetic gain was not significantly different from selection based on GEBVs in the long term. The results of the 3‐ and 6‐generation training populations were all significant in the last generation.

The commercial genetic level was generally higher or equal at all times for criteria looking further ahead into the future than the GEBV (Figure 1D). Generally, across all criteria, the larger the training population, the more prominent the difference in the genetic level compared to GEBV‐based selection. The commercial genetic gain was not significantly different from GEBV selection when using MSV criteria and only the current generation in the training population. When utilising 3 or 6 generations in the training population, all MSV‐considering criteria resulted in significantly higher gains from generation 8 or earlier onward.

### 3.2 Genetic Diversity

**FIGURE 2:**
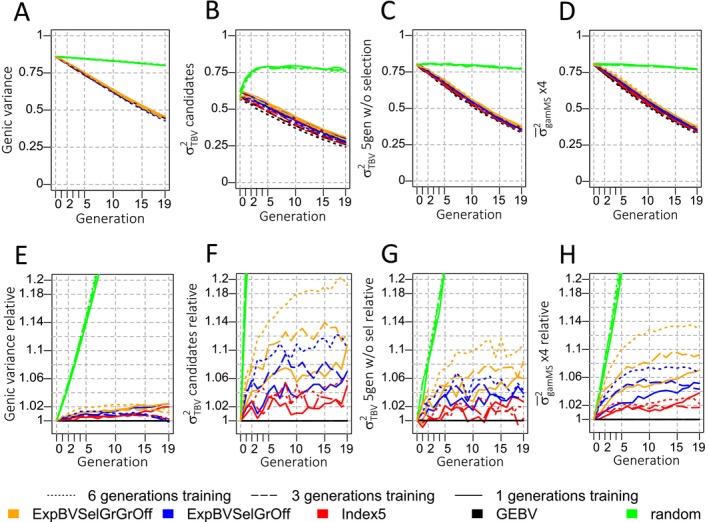
Evolution of measures of different variance metrics: (A) the genic variance in the selection candidates (metric 5), (B) the genetic variance of selection candidates (metric 6), (C) the genetic variance after 5 rounds of random mating without selection (metric 8), (D) the average true gametic Mendelian sampling variance of the selection candidates multiplied by four (metric 7). Plots E–H show the variance metrics expressed as ratios compared to the variance observed in GEBV selection scenarios with the same training population size. Ratios were calculated following this style: $\frac{{\mathrm{VAR}}_{\mathrm{pop}\;{\text{crit}}_{{\mathrm{gen}}_{t}}}}{{\mathrm{VAR}}_{\mathrm{pop}\;{\text{GEBV}}_{{\mathrm{gen}}_{t}}}}$. A ratio of 1.05 should be understood as 1.05 times, or 105%, the variance observed in scenarios using GEBVs as the selection criterion. [Colour figure can be viewed at wileyonlinelibrary.com]

The retained genetic and genic variances were generally larger if the criteria planned further ahead (Figure 2). When including the gametic MSV of animals of the current or future generation in selection criteria, the variance among breeding values of selection candidates showed the largest difference compared to GEBV selection‐based scenarios (Figure 2F). The lowest but still favourable difference to variances observed in GEBV scenarios was observed for the genic variance (Figure 2E). Generally, the larger the training population, the greater the variance that was retained.

The ratio of the genic variance increased until approximately generation 10 and decreased afterward for the 3‐ and 6‐generation training populations (Figure 2E). When using 1 generation in the training set, the ratio of the genic variance rose continuously for MSV‐considering criteria. In generation 5, all MSV criteria and training population size combinations showed significantly higher genic variances. In generation 19, all MSV criteria with a 1‐generation training population and the ExpBVSelGrGrOff criterion with a 6‐generation training population were significantly different compared to selection based on GEBV.

The influence of considering MSV in selection was much stronger on the genetic variance metrics that consider linkage than on the genic variance. All MSV criteria and training population size combinations were significantly different to GEBV selection in all generations, with a few exceptions for Index5 in some generations (where the red lines are close to the black ones in plots F and G in Figure 2). The average gametic MSV appears to be the least erratic (Figure 2H).

The highest variances were observed in the ‘random’ selection scenarios. Since no selection force targeted frequencies of beneficial alleles, the slow decrease of the variance as can be seen in plots A–D in Figure 2 can be attributed to genetic drift alone. The initial increase in genetic variance of the selection candidates for the ‘random’ selection scenarios is caused by the absence of selection pressure after selection during the burn‐in period of 10 generations before the 19 generations shown in Figure 2, i.e., recovery of decreased variance due to the Bulmer effect.

**FIGURE 3:**
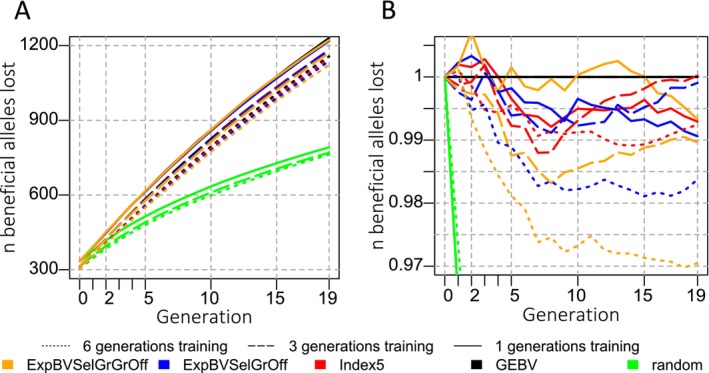
Evolution of the number of beneficial alleles that got lost (metric 4): (A) number of lost beneficial alleles, (B) the number of beneficial alleles lost expressed as a ratio relative to the levels observed when selecting based on GEBV. The ratio is calculated as $\frac{{\bar{n\;\text{beneficial lost}}}_{\mathrm{pop}\;{\text{crit}}_{{\mathrm{gen}}_{t}}}-{\bar{n\;\text{beneficial lost}}}_{\mathrm{pop}\;{\text{GEBV}}_{{\mathrm{gen}}_{0}}}}{{\bar{n\;\text{beneficial lost}}}_{\mathrm{pop}\;{\text{GEBV}}_{{\mathrm{gen}}_{t}}}-{\bar{n\;\text{beneficial lost}}}_{\mathrm{pop}\;{\text{GEBV}}_{{\mathrm{gen}}_{0}}}}$. A ratio of 0.99 means that 0.99 times as many beneficial alleles were lost when selecting with the criterion in question compared to selection based on GEBVs. This is identical to 1% lower loss. [Colour figure can be viewed at wileyonlinelibrary.com]

Longer planning horizons and larger training populations generally resulted in fewer beneficial alleles being lost compared to GEBV selection scenarios (Figure 3). When selection was random, the fewest beneficial alleles were lost. The loss of apparently more beneficial alleles in some scenarios in early generations (generations 1 to 3 in Figure 3B) was not significant. In generation 19, only the ExpBVSelGrOff criterion with the 6‐generation training population and the ExpBVSelGrGrOff criterion with the 3‐ and 6‐generation training populations retained significantly more beneficial alleles.

The inbreeding level measured as the average IBD homozygosity in selection candidates was also much lower when selection was random (Table 2, standard errors in Table ST1). With the exception of scenarios using 1 generation of training, the inbreeding levels achieved with criteria planning further ahead were slightly higher than their GEBV counterparts (Table 2). Inbreeding levels were generally higher if the training population was smaller.

**TABLE 2:** Average IBD homozygosity in selection candidates (metric 3). Standard errors of the mean for inbreeding levels are shown in Table ST1.

		Gen0	Gen1	Gen5	Gen19
1‐Generation training population	Random	0.112	0.116	0.128	0.175
	GEBV	0.112	0.125	0.184	0.383
	Index5	0.112	0.126	0.182	0.378
	ExpBVSelGrOff	0.112	0.126	0.181	0.377
	ExpBVSelGrGrOff	0.112	0.125	0.182	0.378
3‐Generation training population	Random	0.106	0.110	0.122	0.169
	GEBV	0.106	0.120	0.175	0.367
	Index5	0.106	0.120	0.175	0.371
	ExpBVSelGrOff	0.106	0.120	0.174	0.374
	ExpBVSelGrGrOff	0.106	0.120	0.173	0.372
6‐Generation training population	Random	0.103	0.106	0.118	0.165
	GEBV	0.103	0.116	0.169	0.360
	Index5	0.103	0.116	0.168	0.360
	ExpBVSelGrOff	0.103	0.115	0.168	0.364
	ExpBVSelGrGrOff	0.103	0.115	0.167	0.366

### 3.3 Accuracy and Similarity of Selection Decisions

Generally, there is a big overlap between selected animals based on GEBVs and criteria that include gametic MSV. This overlap diminishes as the criterion plans further ahead (Table 3, standard errors in Table ST2). The overlap between decisions was larger for females than for males. The reference population size had an additional influence on the overlap with larger training populations resulting in less overlap in selected animals.

**TABLE 3:** Fraction of animals that are selected based on the criterion that would have also been selected based on GEBV (metric 14). Standard errors are provided in Table ST2.

		Males			Females		
		Gen1	Gen5	Gen19	Gen1	Gen5	Gen19
1‐Generation training population	Random	0.031	0.025	0.036	0.334	0.333	0.335
	GEBV	1	1	1	1	1	1
	Index5	0.897	0.905	0.897	0.952	0.953	0.952
	ExpBVSelGrOff	0.829	0.827	0.815	0.918	0.915	0.910
	ExpBVSelGrGrOff	0.782	0.780	0.769	0.886	0.88	0.876
3‐Generation training population	Random	0.035	0.029	0.032	0.335	0.335	0.335
	GEBV	1	1	1	1	1	1
	Index5	0.877	0.882	0.878	0.945	0.943	0.942
	ExpBVSelGrOff	0.802	0.786	0.783	0.899	0.894	0.891
	ExpBVSelGrGrOff	0.745	0.737	0.716	0.864	0.857	0.849
6‐Generation training population	Random	0.033	0.033	0.028	0.335	0.336	0.335
	GEBV	1	1	1	1	1	1
	Index5	0.871	0.875	0.88	0.939	0.939	0.940
	ExpBVSelGrOff	0.775	0.771	0.778	0.890	0.888	0.882
	ExpBVSelGrGrOff	0.714	0.711	0.676	0.854	0.843	0.829

The training population size directly influences the accuracy of GEBVs which we also observed in this study. The average prediction accuracy for the estimated Mendelian sampling terms, measured as the average correlation between GEBVs and TBVs within full‐sib families (metric 11), was approximately 0.5, 0.6 and 0.65 for 1‐, 3‐ and 6‐generation training populations (Table 4, standard errors in Table ST5). Generally, the prediction accuracy increased over time for random selection but decreased for all other criteria. Interestingly, the prediction accuracies of Mendelian sampling terms differed more from those of GEBV when the selection criterion looked more generations ahead. It even increased slightly from generation 1 to generation 5 for the 3‐ and 6‐generation training populations scenarios. In comparison to the accuracy of Mendelian sampling terms, the prediction accuracy for GEBVs for the selection candidates (metric 10) was higher, at approximately 0.63, 0.68 and 0.72 for the 1, 3 and 6 generations included in the training population (Table ST3). The prediction accuracy for all animals in the training population (metric 9) was approximately 0.63, 0.85 and 0.95 for the 1, 3 and 6 generations included in the training population (Table ST4).

**TABLE 4:** Genomic prediction accuracy of Mendelian sampling terms as the average correlation of true to estimated breeding values within litters (metric 11). Standard errors are shown in Table ST5.

		Gen1	Gen5	Gen19
1‐Generation training population	Random	0.512	0.522	0.528
	GEBV	0.502	0.484	0.424
	Index5	0.507	0.501	0.431
	ExpBVSelGrOff	0.508	0.497	0.438
	ExpBVSelGrGrOff	0.511	0.507	0.446
3‐Generation training population	Random	0.601	0.621	0.643
	GEBV	0.594	0.587	0.531
	Index5	0.603	0.598	0.545
	ExpBVSelGrOff	0.609	0.611	0.557
	ExpBVSelGrGrOff	0.612	0.621	0.576
6‐Generation training population	Random	0.654	0.682	0.712
	GEBV	0.652	0.643	0.605
	Index5	0.658	0.655	0.623
	ExpBVSelGrOff	0.665	0.669	0.634
	ExpBVSelGrGrOff	0.669	0.682	0.649

The accuracies of estimated gametic MSV (Table 5, standard errors in Table ST6) developed similarly as the genomic prediction accuracies. However, with values of approximately 0.26, 0.41 and 0.5 for 1, 3 and 6 generations in the training population, respectively, the accuracy for the prediction of gametic MSV was lower than the accuracy for predicting breeding values.

**TABLE 5:** Correlation of true with estimated gametic MSV in selection candidates (metric 12). Standard errors are shown in Table ST6.

		Gen1	Gen5	Gen19
1‐Generation training population	Random	0.279	0.284	0.305
	GEBV	0.264	0.253	0.218
	Index5	0.262	0.266	0.239
	ExpBVSelGrOff	0.264	0.274	0.248
	ExpBVSelGrGrOff	0.274	0.284	0.256
3‐Generation training population	Random	0.409	0.439	0.475
	GEBV	0.405	0.401	0.352
	Index5	0.413	0.409	0.378
	ExpBVSelGrOff	0.417	0.439	0.396
	ExpBVSelGrGrOff	0.421	0.451	0.423
6‐Generation training population	Random	0.503	0.536	0.582
	GEBV	0.500	0.486	0.451
	Index5	0.501	0.504	0.471
	ExpBVSelGrOff	0.516	0.530	0.506
	ExpBVSelGrGrOff	0.524	0.551	0.547

The correlation between the selection candidates' inbreeding level based on method 1 of VanRaden (2008) and the estimated gametic MSV (metric 15) was negative and weak (down to −0.3; Table 6, standard errors in Table ST7). The correlation became stronger in later generations, and this effect seemed to be slightly more pronounced with smaller training populations. Additionally, the time horizon of a criterion also seemed to have a small effect. The correlation between true IBD homozygosity and the true gametic MSV developed similarly but was approximately 0.02 to 0.04 lower (results not shown).

**TABLE 6:** Correlation of inbreeding level based on method 1 of VanRaden (2008) with estimated gametic MSV in selection candidates (metric 15). Standard errors are shown in Table ST7.

		Gen1	Gen5	Gen19
1‐Generation training population	Random	−0.239	−0.229	−0.223
	GEBV	−0.247	−0.257	−0.292
	Index5	−0.249	−0.259	−0.294
	ExpBVSelGrOff	−0.251	−0.260	−0.295
	ExpBVSelGrGrOff	−0.248	−0.263	−0.296
3‐Generation training population	Random	−0.229	−0.219	−0.210
	GEBV	−0.248	−0.246	−0.275
	Index5	−0.246	−0.252	−0.280
	ExpBVSelGrOff	−0.247	−0.254	−0.291
	ExpBVSelGrGrOff	−0.253	−0.267	−0.300
6‐Generation training population	Random	−0.235	−0.217	−0.211
	GEBV	−0.248	−0.251	−0.268
	Index5	−0.246	−0.250	−0.266
	ExpBVSelGrOff	−0.240	−0.261	−0.285
	ExpBVSelGrGrOff	−0.255	−0.263	−0.297

The ratio of the average estimated gametic MSV to the variance of GEBVs expected within full‐sib families was smaller than 0.5 (approximately 0.39, 0.45 and 0.47 for 1, 3 and 6 generations in the training population), i.e., shrinkage affects the estimated gametic MSV relatively more than GEBVs when the training population is smaller (Table 7, standard errors in Table ST8). Interestingly, the ratio remained roughly the same over generations or even increased slightly (1‐ and 3‐generation training populations), except when selection was random, in which case the ratio decreased slightly. The ratio was smaller for criteria with a longer planning horizon. This pattern was also observed for the ratio of true gametic MSV to true full‐sib variance, although less pronounced (Table 7).

**TABLE 7:** Ratio of average gametic MSV over variance expected in full‐sib families corrected by inbreeding level (metric 13). Corresponding standard errors of the mean are provided in Table ST8.

		Ratio estimated variances			Ratio true variances		
		Gen1	Gen5	Gen19	Gen1	Gen5	Gen19
1‐Generation training population	Random	0.394	0.395	0.387	0.501	0.500	0.499
	GEBV	0.391	0.397	0.417	0.498	0.495	0.501
	Index5	0.389	0.396	0.413	0.493	0.491	0.496
	ExpBVSelGrOff	0.386	0.395	0.415	0.493	0.493	0.493
	ExpBVSelGrGrOff	0.390	0.396	0.415	0.495	0.494	0.491
3‐Generation training population	Random	0.455	0.452	0.449	0.501	0.502	0.499
	GEBV	0.457	0.456	0.468	0.501	0.503	0.505
	Index5	0.450	0.451	0.459	0.499	0.499	0.500
	ExpBVSelGrOff	0.451	0.446	0.455	0.498	0.494	0.502
	ExpBVSelGrGrOff	0.447	0.446	0.453	0.497	0.495	0.494
6‐Generation training population	Random	0.483	0.473	0.474	0.507	0.501	0.503
	GEBV	0.477	0.478	0.479	0.500	0.503	0.505
	Index5	0.472	0.465	0.467	0.499	0.497	0.496
	ExpBVSelGrOff	0.466	0.463	0.463	0.490	0.492	0.493
	ExpBVSelGrGrOff	0.465	0.459	0.464	0.492	0.489	0.492

## 4 Discussion

We tested the impact of considering current and future gametic MSVs on selection decisions in a simplified pig breeding scheme when basing computations on estimated SNP effects rather than known ones. While conventional genetic gain was initially lower, the commercial genetic gain was always higher or at least equal compared to GEBV‐based selection, although the advantage was relatively small. In addition, considering MSV in selection decisions resulted in higher genomic prediction accuracies and retained more trait‐affecting diversity. The higher the genomic prediction accuracy, the greater the benefit of including MSV in selection decisions.

### 4.1 Effect on Gain

Accounting for the gametic MSV of selection candidates (Index5) and their descendants (ExpBVSelGrOff and ExpBVSelGrGrOff) initially resulted in lower population average breeding values compared to GEBV‐based selection (Figure 1). This is because MSV‐considering criteria maximise the population average breeding value in a future generation. Until that generation is reached, the population average breeding value of a population selected with an MSV‐considering criterion is lower than if that population was selected based on GEBVs. For example, the ExpBVSelGrGrOff criterion aims at maximising the population average breeding value in four generations. In contrast to the expectation, Figure 1C shows clearly that the genetic level after four generations is lower than in populations selected based on GEBVs. Apart from the effect of imperfect SNP effect estimation, this is because for actual maximisation of gain in generation 4, one has to plan ahead by four generations in generation 0 (ExpBVSelGrGrOff), three generations in generation 1 (ExpBVSelGrOff), two generations in generation 2 (Index5) and one generation in generation 3 (GEBV). However, we used the same criterion in every generation. From a breeding perspective, this means accepting a lower population average breeding value up to the planning horizon of the respective selection criterion in every generation.

Figure 1D shows that the commercial genetic level was however higher or at least the same as in populations selected based on GEBVs. This is mainly due to greater genetic variances when selecting with MSV‐criteria. Thus, the competitiveness of breeding programs does not seem to be compromised when using either of the MSV‐considering selection criteria. For reference, Bijma, Wientjes, and Calus (2020) predicted an increase in response to selection of 0.6%–1.3% for selected fractions of 5%–10% for populations with a coefficient of variation (CV) of the gametic Mendelian sampling standard deviation ($\frac{\mathrm{sd}\left(\sqrt{\text{gamMSV}}\right)}{\mathrm{av}\left(\sqrt{\text{gamMSV}}\right)}$) of 0.1 (see their Tables 2 and 4). The CV in our simulation was approximately 0.11–0.12. The proportion of selected candidates corresponding to the applied selection intensities (Table 1) corresponds to 7% for the selection of AI boars (used in Index5) and 12% for population improvement (also used in ExpBVSelGrOff and ExpBVSelGrGrOff). With at most 1% more gain in Index5 scenarios, the increase in commercial genetic gain seems to fall slightly short of the prediction of Bijma, Wientjes, and Calus (2020).

### 4.2 Effect on Diversity

Considering MSV in selection decisions resulted in up to 20% higher genetic variance among the selection candidates compared to selecting based on GEBVs only (Figure 2). This effect was more pronounced for criteria with a longer planning horizon and for better genomic prediction accuracies. This is because the emphasis on diversity is greater when breeding values are to be maximised further in the future. The positive effect of higher prediction accuracies can be attributed to the fact that QTL effects, or the sum of QTL effects on a haplotype, are estimated more precisely based on more accurately estimated SNP effects which in turn results in more accurate estimates of MSV. Thus, with an increasing reference population size, the benefit of using MSV is expected to rise, given that SNP effects will be estimated more accurately (Meuwissen, Hayes, and Goddard 2001; Wimmer et al. 2013). For clarification, in our study, the expansion in training population size is accompanied by an increase in depth. The results may differ if training sets of different sizes but the same depths are utilised. We chose to enlarge the training set size by including more information from previous generations, i.e., increasing the depth, because this is the most practical option for real breeding programs. We expect that a rise in size rather than depth would improve accuracy for selection candidates to a greater extent. This is because the linkage disequilibrium and allele frequencies of the training set can be expected to be more similar to those of the selection candidates with fewer separating recombination events between the training and prediction sets.

The genetic variance of selection candidates is the most relevant aspect for practical breeders as it represents the variation out of which animals for production or animals that produce production animals can be selected. This variance, however, is highly influenced by the variance of parent average breeding values, which is reduced if the parents are selected. The reduction is strongest when selecting based on GEBVs and weaker the more generations a criterion is planning ahead. The selection‐induced change in genetic variance due to changes in linkage disequilibrium, i.e., the Bulmer effect (Walsh and Lynch 2018, p. 553), is however not permanent and can be reversed when selection pressure is loosened. This is why the genetic variances in the scenarios with random selection, after genomic selection in the 10‐generation‐long burn‐in period, increase for approximately the first four generations (Figure 2B). Thus, we did not deem the genetic variance of selection candidates a relevant metric for judging long‐term sustainable variance. We used five generations of random mating without selection with a large population size to pragmatically obtain the selection‐unaffected genetic variance.

Since the genetic variances obtained with this method for the ‘random’ selection scenario do not demonstrate the initial increase compared to the genetic variance of selection candidates, the variance reduction due to the Bulmer effect appears to have been successfully removed (Figure 2C). This somewhat pragmatic way of correcting for reduced parent average variances may also result in the gradual loss of beneficial linkage disequilibrium (LD) between beneficial loci. The average true gametic MSV multiplied by 4 should account for the reduction in parent average variance without changes in the within‐chromosome LD structure. This metric also does not show the initial increase after the burn‐in phase as observed for the variance of TBVs of candidates in the random scenario (compare B and D in Figure 2). In line with reports by Lara et al. (2022), this means that selection mainly induces negative LD, or covariance, between beneficial alleles on different chromosomes in the parent average breeding values. The gametic MSV multiplied by 4 generally exhibited a larger difference to the variance observed in GEBV scenarios with at most 14% more genetic variance when selecting with ExpBVSelGrGrOff (Figure 2G,H). Note that we refer to any kind of disequilibrium as ‘linkage disequilibrium’ in this study, even if the underlying cause is not necessarily physical linkage.

With at most 2% benefit, including MSV in selection decisions did not have as pronounced a benefit on the genic variance as on our genetic variance metrics (compare plot E to F–H of Figure 2). The total additive genetic variance ($\sigma_{A}^{2}$) differs from the genic variance due to LD and deviations from expected genotype frequencies under Hardy–Weinberg equilibrium (Wang, Caballero, and Hill 1998). Thus, the total additive genetic variance can be expressed as the sum of the genic variance ($\sigma_{a}^{2}$) and the contribution of disequilibrium (d) as $\sigma_{A}^{2}=\sigma_{a}^{2}+d$ according to Walsh and Lynch (2018), p. 550. Considering that we only find a small benefit regarding the genic variance ($\sigma_{a}^{2}=2\sum_{i=1}^{N}p_{i}\left(1-p_{i}\right)\alpha_{i}^{2}$) relative to the benefit for the total variance, this means that the beneficial difference in total additive genetic variance is mainly due to less negative LD and only to a lesser degree due to shifting the frequencies of beneficial alleles closer to 50%. This is in line with the findings of other simulation studies (Allier et al. 2019; Niehoff et al. 2024). To prove that the variance is increased by ‘less negative LD’ and not by ‘more positive LD’, we calculated the ratio of four times the gametic MSV over the genic variance. Under linkage equilibrium, the ratio is 1 since the gametic MSV is a quarter of the genic variance. We observed that this ratio was always lower than 1. This ratio also decreased over generations but was always higher for MSV‐considering criteria compared to GEBV‐based selection (Table ST9).

We also found that fewer beneficial alleles were lost from the population when considering the MSV in selection decisions, a finding which is in line with reports from literature (Allier et al. 2019; Musa and Reinsch 2023; Niehoff et al. 2024; Sanchez et al. 2023). This suggests that the variance component of the selection criteria helps to keep beneficial variants in the population. To increase the gametic MSV, it is more useful to increase the frequency of rare alleles than that of common ones. This is because when adding one copy of the minor allele, the genetic variance caused by a locus ($2p\left(1-p\right)\alpha^{2}$), and by extension the average gametic MSV, increases more for a locus with a low MAF than a high one.

In contrast to simulation studies in which breeding values and gametic variances are calculated based on the underlying true allele substitution effect (Musa and Reinsch 2023; Niehoff et al. 2024), we did not detect a beneficial effect on inbreeding levels, i.e., neutral diversity, or non‐trait affecting diversity. In this paper, we found a slight increase in inbreeding levels after 19 generations compared to selection based on GEBVs in scenarios with 3‐ and 6‐generation training populations for genomic prediction (Table 2). This was unexpected, since the MSV is expected to be perfectly inversely correlated to the inbreeding and kinship of founders under the infinitesimal model (see Niehoff, ten Napel, and Calus 2024), and because we identified a weak but negative correlation between gametic MSV and inbreeding levels (Table 6). While the difference between inbreeding rates per generation ($\left(\mathrm{av}\;{\text{inbreeding}}_{\mathrm{gen}\;t}-\mathrm{av}\;{\text{inbreeding}}_{\mathrm{gen}\;0}\right)/t$) with 1.353% and 1.387% for GEBV and ExpBVSelGrGrOff over the 19 generations with 6 generations in the training population is small, it is still useful to hypothesize why we see a slight negative effect on neutral diversity (inbreeding rates not shown elsewhere).

It may be that by selecting based on estimated MSV, not only those animals with a truly high MSV are selected but also those for which the estimate of the MSV is higher. SNPs that segregate with a higher MAF will be estimated more accurately. Their estimated effect will thus be less shrunken toward 0 than is the case for SNPs with a low MAF. Consequently, an animal that is heterozygous at high MAF loci will have a higher estimate of the MSV than one that is heterozygous at low MAF loci, even if the heterozygosity of both is identical. The lower the MAF at a locus, the lesser the average per locus relationship of a carrier of a rare allele to the population. In other words, mating a heterozygous animal to the population results in less homozygous offspring if the MAF in the population is lower (for a more elaborate explanation, see ‘Inheritance of gametic variability’ in the discussion of Bijma, Wientjes, and Calus (2020)). This suggests stronger shrinkage of MSV of animals with a low relationship to the training population. Thus, more related animals may be selected by basing selection decisions on estimated properties.

Another reason may be that the contribution of families to the group of selected candidates varies more with selection based on MSV‐considering criteria. When using truncation selection based on GEBV, relatively fewer progeny of parents with a lower GEBV will be selected since these have, on average, a lower GEBV as well (Falconer and Mackay 1996). This leads to a greater variation of contributions between families. An increased variance in genetic contributions raises the inbreeding rate (Falconer and Mackay 1996, p. 68). Considering the MSV in addition to breeding values caused an increase in parent average variance, i.e., the difference in GEBV between the best and worst selected animals is enlarged. We calculated the MSV criteria under the assumption of an equal contribution from every parent, which was not realised in the simulation. In this study, the selection of offspring was not based on their GEBVs, but the GEBV has a strong influence on the value of MSV‐considering criteria (see Table 7 in Niehoff, ten Napel, and Calus (2024)). For simplicity, we utilised the GEBV to hypothesize about variation in contributions here.

It is not clear what is causing the unexpected small increase in inbreeding. In practical applications, however, the impact could be mitigated by adding lowly related animals to the training population (first reason), or by imposing a constraint on the maximum contribution of families (second reason).

The decrease in variance over generations can mainly be explained by the faster increase of the average inbreeding level in scenarios with directional selection compared to random selection (Table 2 and Figure 2A–D). To a smaller degree, it can also be explained by moving beneficial alleles closer to fixation with selection. This is because the genic variances were approximately 0.444, 0.436 and 0.429 in the last generation for GEBV selection when using 1, 3 or 6 generations in the training population, respectively, even though the homozygosity levels (Table 2) and the loss of beneficial alleles (Figure 3A) were lowest with the largest training population and highest with the smallest one. In other words, the inbreeding level was not predictive of the genic variance when comparing the outcomes of different training population sizes.

### 4.3 Accuracy and Similarity of Selection Decisions

The lowest accuracies were found for the prediction of the Mendelian sampling term, i.e., for within‐family prediction (Table 4). This is because this metric removes the predictive power the genetic trend over generations (Table ST4) and the parent average breeding value have on the breeding values of the candidates (Table ST3) (Werner et al. 2020). In the following section, we will only focus on this metric.

Due to decreasing genetic variances, the heritability decreased as well. The heritability ($V_{\mathrm{TBV}}/\left(V_{\mathrm{TBV}}+V_{E}\right)$) was 0.3 at the start of the burn‐in period, approximately 0.21 in generation 0 of the breeding program and approximately 0.1 in generation 19 in all scenarios with selection. For completeness, when expressed with the genic variance ($V_{\text{genic}}/\left(V_{\text{genic}}+V_{E}\right)$), heritability decreased as well, namely from 0.3 to 0.27 in generation 0, and to 0.16 in generation 19. Decreasing heritability values have also been reported for a real pig breeding program (Hidalgo et al. 2020). We found increasing prediction accuracies for the Mendelian sampling term over generations for the random selection scenario and decreasing accuracies for all other criteria (Table 4). We hypothesize that the decrease in heritability is the main reason for lower prediction accuracies in generation 19 compared to generation 1 for non‐random selection.

When including the MSV in the selection criteria, the accuracy was always higher than if the selection was based on GEBVs (see Table 4). This apparent positive effect on prediction accuracies when considering MSV may be because of two reasons. First, considering the MSV in selection means that some weight is placed on selecting animals so that the offspring variance is increased. Higher variances imply that the average difference between two offspring individuals is larger. Thus, SNP effects can be estimated more accurately since it is easier to attribute differences in observed phenotypes to genotypic differences. For reference, we found a positive correlation between within‐litter accuracy and within‐litter genetic variance (spearman rank correlation 0.35, 0.41 and 0.45 in generation 0 for 1, 3 and 6 generations in the training population, results not shown elsewhere). A second reason may be that by considering the MSV in selection decisions, the variance of parent average breeding values is reduced less than would be the case with GEBV‐based selection. This increases the covariance of breeding values of full‐sib offspring. In other words, the effects of alleles that segregate across families may be estimated more accurately.

To estimate the difference in shrinkage acting on GEBVs compared to gametic MSV, we calculated the ratio of the average gametic MSV over the average variance of full‐sib offspring ($\frac{{\bar{\sigma }}_{\text{gamMS}}^{2}}{{\bar{\sigma }}_{\mathrm{FS}}^{2}}$). If the shrinkage acting on GEBVs is the same as that acting on MSV, the ratio between both is expected to be 0.5. This value was only observed in some scenarios when using the true QTL effect. With estimated SNP effects, the smaller the training population, the lower the ratio (Table 7). This indicates that the shrinkage acting on gametic MSV is systematically higher than that on GEBVs. Note that all animals have their own phenotypic observations in the breeding value estimation in this study which increases the accuracies of their corresponding GEBVs, and thus decreases the shrinkage of GEBVs. This is not the case for the estimated gametic MSV as no phenotypes of gametes are available. For example, since all QTLs are expressed in the phenotype, all QTLs contribute to the GEBV accuracy, but only QTLs in LD with genotyped SNPs contribute to the accuracy of estimated MSV. Without phenotypes of selection candidates, we expect that the shrinkage of GEBV and estimated gametic MSV would be somewhat the same, which would result in the variance ratio being closer to 0.5. This expectation needs to be validated in further research. In addition, we corrected the gametic MSV and the deviation from the parent average breeding value with the inbreeding level. In this paper, the inbreeding level for the ratio was obtained from the genomic relationship matrix according to method 1 of VanRaden (2008). We acknowledge that this measure may not be the best one for representing the true homozygosity level. For example, the correlation between the inbreeding level obtained with the genomic relationship matrix and the true IBD inbreeding level was approximately 0.82 in this work. The correction for inbreeding in the ratio was carried out to avoid deviations from 0.5 purely due to drastic changes in the inbreeding level from the parent generation to the offspring. If the inbreeding level does not change drastically, it may be more convenient for real data sets to calculate the ratio without the inbreeding correction.

The fact that estimated MSV are shrunken more than GEBVs means that predicted variances have a relatively lower impact on selection decisions than the GEBV. This is reflected in selection decisions becoming more similar to selection decisions based purely on GEBVs for smaller variance ratios (see Tables 3 and 7).

The ratio of average gametic MSV over average full‐sib variance more generally represents the ratio of indirectly predicted next‐generation average gametic MSV to the variance of Mendelian sampling terms in the current generation, and thus may also be calculated for species that do not have the full‐sib family structure as in pig breeding. We see this ratio and the correlation between true and estimated gametic MSV as two critical parameters for judging the effectiveness of using one of the MSV‐considering criteria in a breeding program. While the ratio can easily be estimated, estimating the correlation between true and estimated gametic MSV is impossible since true allele effect are never known. Thus, an alternative approach that has been utilised in empirical studies is to compare the predicted gametic MSV with the GEBV variance of many offspring of an individual.

For reference, reports of the correlation between predicted and observed variances for dairy cattle for German Holstein (Segelke et al. 2014), US Holstein and Jersey (Santos et al. 2019), Dutch Holstein (Linders 2020) and French Holstein (Hozé et al. 2022) show correlations from 0.14 up to 0.98 depending on the trait and the number of offspring per sire used for validation. Unfortunately, those works do not indicate the accuracy of the GEBV. Based on reports from plant breeding research, the accuracy for predicting progeny variances is lower than the accuracy for GEBVs in various species (Adeyemo and Bernardo 2019; Neyhart and Smith 2019; Oget‐Ebrad et al. 2024; Wolfe et al. 2021) which is in line with our findings (compare Table 4 with Table 5). For comparison, the highest correlation between true and estimated gametic MSV in our simulation study was 0.55 (Table 5).

### 4.4 Potential to Improve the Prediction Accuracy of Progeny Variances

The accuracy of GEBVs depends on the LD between QTL and SNPs used for genotyping (Goddard 2009; Habier, Fernando, and Dekkers 2007; Habier, Fernando, and Garrick 2013). The association between markers and QTLs decays through recombination, which is why genomic prediction accuracies decrease when more generations (meiotic events) separate animals from the genomic prediction reference set (e.g., Dekkers, Su, and Cheng 2021; Habier, Fernando, and Dekkers 2007; Hidalgo et al. 2021; Hollifield et al. 2021; Meuwissen, Hayes, and Goddard 2001; Pszczola et al. 2012). Similarly, the SNP effect estimates of consecutive evaluations are strongly correlated, but this correlation decreases rapidly when the evaluated cohorts are more distant (Liu et al. 2011; Richter et al. 2024). The prediction of future progeny variances can be understood as the forward prediction of breeding values of more detached animals. Thus, prediction models whose accuracies can be expected to be impacted less strongly by LD decay, such as approaches that directly incorporate the prior information of some major loci from, e.g., genome‐wide association studies (Meuwissen, Eikje, and Gjuvsland 2024), might result in higher and more sustained accuracies of gametic MSV over generations.

Variances predicted based on estimated SNP effects are, strictly speaking, only reflecting the expected variances of GEBVs in the future, but not the true genetic variances, given that no new phenotypic information is added to the prediction model. Consequently, the within‐family variances predicted based on estimated SNP effects underestimate the variance of TBVs because the variance due to the prediction error is missing ($V_{\mathrm{TBV}}=V_{\mathrm{EBV}}+\mathrm{PEV}$). We did not incorporate the prediction error variance (PEV) into the prediction of future progeny variances because the variance of EBV represents the variance that is explainable by the model. Predicting the variances of GEBVs avoids having to make assumptions about the accuracy, i.e., the square root of the predicted variances of GEBVs can be multiplied with the selection intensity directly to obtain the selection differential. In contrast, if the PEV had been included in the prediction of future progeny variances, we would also require knowledge about the selection accuracy in the generations of descendants, and this accuracy may differ per family.

To accurately predict the variance of the TBVs of the next generation, the variances of the parent average TBVs of the families and the average within‐family variance need to be summed (e.g., Shepherd and Kinghorn 1998; $\begin{array}{c} V_{\mathrm{TBV}}=V_{\mathrm{PA}\;\mathrm{TBV}}+{\bar{V}}_{\text{within}-\text{family}\;\mathrm{TBV}}=V_{\mathrm{PA}\;\mathrm{EBV}}+{\mathrm{PEV}}_{\mathrm{PA}\;\mathrm{EBV}}+{\bar{V}}_{\text{within}-\text{family}\;\mathrm{EBV}}+{\bar{\mathrm{PEV}}}_{\text{within}-\text{family}\;\mathrm{EBV}} \end{array}$). Note that this does not refer to the within‐family variance as this variance can be larger than the true within‐family variance, even if only one family is selected, because of the PEV of the family mean. For the same reason, the predicted variance of the TBVs of F1 offspring produced from two identical inbred lines (i.e., both parents and offspring are identical) would be larger than 0 as long as ${\mathrm{PEV}}_{\mathrm{PA}\;\mathrm{EBV}}>0$, i.e., as long as accuracy is imperfect. In this case, the term ‘genetic variance’ may be somewhat misleading. This variance can perhaps instead be thought of as representing a ‘range’ within which one would expect the TBV of offspring to be located, rather than an actual genetic variance due to segregation. Lehermeier, Teyssedre, and Schön (2017) attempted to incorporate the uncertainty with which SNP effects are estimated, which represents the PEV (not explicitly mentioned by the authors) in the prediction of within‐family variances. This seems to be partly motivated by the fact that variances based on estimated SNP effects are lower than true progeny variances. Lehermeier, Teyssedre, and Schön (2017) call this difference ‘bias’. For reference, the ‘bias’ calculated in the style of Lehermeier, Teyssedre, and Schön (2017) that we observed in our simulation study for gametic MSV is shown in Table 8. This ‘bias’ is to be expected when using the estimated SNP effects of genomic prediction models since these shrink effects, as discussed previously.

**TABLE 8:** Average ‘bias’ for the gametic Mendelian sampling variance according to the definition of Lehermeier, Teyssedre, and Schön (2017) ($\left({\hat{\sigma }}_{\text{gamMS}}^{2}-\sigma_{\text{gamMS}}^{2}\right)/\sigma_{\text{gamMS}}^{2}$).

		Gen1	Gen5	Gen19
1‐Generation training population	Random	−0.707	−0.704	−0.711
	GEBV	−0.717	−0.706	−0.638
	Index5	−0.716	−0.697	−0.642
	ExpBVSelGrOff	−0.716	−0.701	−0.642
	ExpBVSelGrGrOff	−0.712	−0.704	−0.637
3‐Generation training population	Random	−0.572	−0.557	−0.542
	GEBV	−0.557	−0.546	−0.503
	Index5	−0.558	−0.540	−0.501
	ExpBVSelGrOff	−0.555	−0.533	−0.489
	ExpBVSelGrGrOff	−0.553	−0.532	−0.484
6‐Generation training population	Random	−0.429	−0.492	−0.438
	GEBV	−0.427	−0.431	−0.414
	Index5	−0.424	−0.426	−0.397
	ExpBVSelGrOff	−0.417	−0.412	−0.381
	ExpBVSelGrGrOff	−0.414	−0.412	−0.377

Lehermeier, Teyssedre, and Schön (2017) derive the PEV of individual SNPs by calculating the variance of the SNP effect estimates of many post‐burn‐in samples when using Bayesian ridge regression, called the ‘posterior mean variance (PMV)’ method in their paper. Since the posterior variance of GEBV samples represents the PEV (Gao et al. 2018), the posterior distribution of SNP effect estimates can also be employed to infer SNP‐based PEV and prediction error covariance (PEC). In addition to somewhat unbiased estimates of progeny variances, Lehermeier, Teyssedre, and Schön (2017) also report improved prediction accuracies for progeny variances (see their Figure 3). The reader interested in testing the PMV method is referred to the Supporting Information of Allier et al. (2020), who provide relevant R code. Methods to estimate PEV and PEC for SNPs have also been developed in animal breeding research (Aguilar et al. 2019; Gao et al. 2023; Garcia et al. 2022; Gualdrón Duarte et al. 2014), even for single‐step genomic prediction, mainly in the context of reliability estimation. For example, SNP‐based PEV and PEC can be calculated with programs of the BLUPF90 software suite (Garcia et al. 2022) and with the MiXBLUP software suite (Vandenplas and Bonifazi 2024).

To our knowledge, benefits of the PMV method have only been reported in simulation studies (Lehermeier, Teyssedre, and Schön 2017; Oget‐Ebrad et al. 2024). However, studies based on real wheat (Oget‐Ebrad et al. 2024) and cassava (Wolfe et al. 2021) data unexpectedly report lower correlations between estimated and observed progeny variances when the variance estimation is done with the PMV method. The figures from the Wolfe et al. (2021) paper supporting this statement can be found in their Figure S10 and Table S11 in the Excel file of their Supporting Information. Further work is needed to test if incorporating PEV indeed improves progeny variance predictions. Apart from potentially improving the predictive correlation of progeny variances, this method is interesting because utilising prediction errors may also implicitly attribute more variance, and thus importance, to rare alleles, since these are likely estimated with higher uncertainty, i.e., higher PEV (Goddard 2009). Upweighting rare alleles might have the beneficial effect that these variants may be less likely to be lost from the population due to drift.

### 4.5 Practical Implementation

In this study, we only simulated a single trait whereas the breeding goals of actual breeding programs include a combination of multiple traits. When selection is carried out with a selection index, we suggest approximating the allele substitution effects of the index to calculate variances. This is similar to pretending that the selection index is the trait. The allele substitution effect for the index at a particular locus can be calculated as the weighted sum of estimated trait‐specific effects with the selection index weight as the weight, i.e., $\alpha_{\text{index}}=w_{\text{trait}\;1}\alpha_{\text{trait}\;1}+w_{\text{trait}\;2}\alpha_{\text{trait}\;2}+\ldots +w_{\text{trait}\;k}\alpha_{\text{trait}\;k}$ with w as the weight of the trait in the index and α as the estimated allele substitution effect for that trait at the locus in question. Using the allele substitution effects in this manner results in the same prediction of the index variance as if calculated following the method of Equation 7 in Musa and Reinsch (2023).

A question commonly raised by applied geneticists is how maximising genetic gain in a certain future generation works if the weights of the selection index change. Ideally, the selection index weights that are to be applied in, e.g., three generation's time are already known now and should thus be used in the calculation. However, future index weights are unlikely to be available. But in cases where the index weights change and selection has been carried out based on the old weights, we do not see major issues. This is because selection index weights do not change drastically from one generation to another. For example, fertility and maternity traits will always be of high importance for a dam line and the higher retained genetic variance for the traits will be beneficial, even when the importance of the traits decreases or increases by, e.g., 5%.

All MSV‐considering criteria require information about the selection intensity to weigh in the Mendelian sampling standard deviation. In theory, the intensity can be derived based on the fraction of selected animals. In practice, however, this might not be as easy, for example, because the selected parents have varying contributions to the next generation or because of overlapping generations. In that case, an alternative approach is to compute the realised selection intensity by dividing the realised response to selection per generation $\left({\bar{\mathrm{EBV}}}_{\text{selected offspring}}-{\bar{\mathrm{EBV}}}_{\text{parents}}\right)$ with the standard deviation of EBVs of selection candidates in that generation.

We developed a new heuristic selection algorithm with the goal of making good selection decisions while only evaluating a fraction of the entire solution space. By adopting the concept of selecting based on GCA (Falconer and Mackay 1996, pp. 274–276), we managed to make selection decisions for an individual based on values that are expressed for a pair (ExpBVSelGrOff) or a quartet of individuals (ExpBVSelGrGrOff). An algorithmic solution may still be impractical or time‐consuming for some breeding programs. A more efficient alternative may be to consider, e.g., the top 100 animals of a population as testers and calculate values for only this limited number of ‘testcrosses’. The resulting average value may then be used for selection.

The successful application of MSV‐considering criteria requires that all selected animals have the same chance to produce offspring, or that their contributions are optimised based on some other diversity management scheme, e.g. optimum contribution selection (Meuwissen 1997). We believe that the best use of the tested MSV‐considering criteria is in addition to established diversity management tools that, e.g., consider the average relatedness of an animal to a breed. Our recommendation would be to treat the MSV criterion as the breeding value in diversity management. For example, the average ExpBVSelGrGrOff value of a bull assuming random mating to the population, or to the 100 testcrosses as discussed above, should be used in place of the GEBV in optimum contribution software. The software MateSel is able to work with user‐supplied mating‐specific ExpBVSelGrOff values directly (Kinghorn and Kinghorn 2024; see p. 102 in the software instructions).

In this study, we only simulated additive gene actions, but in reality, genetic performance is also influenced by dominance effects and epistatic interactions. Further testing and evaluation in real programs need to be carried out, but we believe that considering the MSV in selection would show positive results in real breeding programs as well. This is because the statistical additive effect of QTLs is somewhat stable over generations. We base this statement on the results of Wientjes et al. (2022), who simulated a genomic breeding program with additive, dominance and epistatic allele effects. The correlation of the true statistical additive effect, or ‘average effect’, between two cohorts separated by 10 generations of selection was high at approximately 0.9 (see their Figure 10).

## 5 Conclusion

We investigated the use of MSVs in a pig breeding scheme when SNP effects are estimated. While commercial genetic gain was never negatively impacted, we confirmed that considering MSVs in selection can lead to higher long‐term gains and greater genetic variances compared to ordinary selection based on GEBV. The larger the reference population for genomic prediction and the more generations were considered in the selection criterion, the greater the benefit. While added benefits may be small, as we observed maximum increases in genetic gain of 2% and in genetic variance of 20% compared to GEBV selection, we found no apparent critical trade‐offs when switching to one of our tested selection methods.

## Conflicts of Interest

TAMN and MPLC would like to acknowledge that they are listed as inventors on a patent application, no. EP23192016.6, pending at the European Patent Office, related to the technology discussed in this paper, specifically the prediction of variances of 2 or more generations ahead and the selection looking 2 or more generations ahead based on predicted variances of descendants. JtN has no conflicts of interest to declare.

## Supporting information


Data S1.

## Data Availability

The data that support the findings of this study are available from the corresponding author upon reasonable request.
