# Supplementary material for: Practical Considerations When Using Mendelian Sampling Variances for Selection Decisions in Genomic Selection Programs
Source: J Anim Breed Genet. 2024 Dec 2;142(4):419–37. doi: 10.1111/jbg.12913 (PMC12149496; doi:10.1111/jbg.12913)
Supplement: Supplementary file 1 — Data S1. [file JBG-142-419-s001.docx]

Supplementary Material

Supplementary Material SM1

Selection algorithm

In hybrid variety development e.g. as in corn or rye, the problem is that very many combinations of parental genotypes are possible to create the best possible genotype, i.e., hybrid variety. Conducting all possible crosses to evaluate the resulting hybrids is often not feasible because of limited resources like field size. Thus, breeders often use so called tester lines which resemble the genetic pool of the crossing partner. A tester may be one or a few dedicated lines or selection candidates of the opposite pool. By crossing all selection candidates to only one or a handful of testers, breeders get a general impression of the selection candidates’ performances in a hybrid variety. The general combining ability (GCA) of a selection candidate may be estimated as deviation of the average performance off all its hybrid offspring genotypes resulting from crosses of the candidate to the testers from the average hybrid performance of all selection candidates (Falconer & Mackay, 1996, p. 274). For simplicity, we will just consider the GCA as the average performance of hybrid offspring of an individual without considering the mean as this is just an intercept in a statical sense.

In this study however, we are investigating the use of new selection criteria (ExpBVSelGrOff and ExpBVSelGrGrOff) in a pig breeding scheme for pure line improvement. There is no dedicated other heterotic pool and thus no obvious tester. Thus, we used other members of the same population that are likely to be in the group of selected individuals as testers. In simple words, the ExpBVSelGrOff criterion was calculated for females in combination with the best males (testers) and vice versa. The ExpBVSelGrGrOff criterion was calculated for females in combination with a number of trios of three of the best individuals of the population (testers) and vice versa. Later, only the individuals with the highest average ExpBVSelGrOff or ExpBVSelGrGrOff values (GCAs) were selected. Based on the number of individuals considered, the ExpBVSelGrOff criterion is analogous to a 2-way hybrid whereas the ExpBVSelGrGrOff criterion is analogous to a 4-way hybrid.

In a first step to attempt to decrease the solution space of sires and dams to select, we preselected the top 400 males and top 800 females based on their Index5 values. That means that 400 x 800 = 320,000 pairs (ExpBVSelGrOff) and (400 x 800)^2^ =102,400,000,000 quartets (ExpBVSelGrGrOff) remain in the solution space, which means a reduction to 22% and 5% of the original number of possibilities for ExpBVSelGrOff and ExpBVSelGrGrOff, respectively.

Starting with those 400 preselected males and 800 preselected females, we iteratively further reduced the number of male and female candidates while evaluating the GCA values of the remaining candidates more and more thoroughly. In every iteration, the number of surplus selection candidates was halved. For instance, the surplus number of males in the first iteration (400-40) was halved, leaving 40+(400-40)/2=220 male candidates for the second iteration (Table 1). In every iteration, we used 4 additional testers for all remaining female selection candidates and 10 additional testers for all remaining male selection candidates. Note that testers for the ExpBVSelGrGrOff criterion are trios. The algorithm stopped once the surplus of selection candidates was lower than 10% of the number of animals that are to be selected. In this study, this means that the algorithm stopped after 8 iterations.

The testers in the first iteration were selected based on their highest GEBV as these animals are likely to be selected based on the ExpBVSelGrOff and ExpBVSelGrGrOff criteria as well. In any further iteration, the animals with the highest GCA values were selected as additional testers. Table 1 shows the number of testers used to calculate GCA values for selection candidates in every iteration. The same testers were used for all selection candidates within sex.

In total, with the strategy of the algorithm as described here, only 26,386 matings or quartets had to be evaluated. This is only 8% and 0.00003% of the number of possibilities within the preselected set of candidates for the ExpBVSelGrOff and ExpBVSelGrGrOff criterion, respectively.

For the ExpBVSelGrOff criterion, only males could be chosen as testers for females and vice versa. The tester trios for the ExpBVSelGrGrOff were sex specific too, i.e., the combination was (focal_male x tester_female1) x (tester_male1 x tester_female2) for males and (focal_female x tester_male1) x (tester_female1 x tester_male2) for females. The order of the three individuals in a tester trio was not optimized. An individual could only be part of one tester trio to avoid double counting. The number of males and females used for all tester trios is shown in Table 2. Since the number of male individuals required for tester trios to evaluate females exceeds the number of remaining male selection candidates in the last two iterations, the males with the highest ever recorded ExpBVSelGrGrOff values that were discarded in earlier iterations were used. This choice is somewhat arbitrary. An alternative strategy for future study could be to reuse some previously selected males with very high average ExpBVSelGrGrOff values to reflect their higher genetic contribution to later generations.

| Iteration | Male selection candidates | Female selection candidates | Number of tester for males | Number of tester for females |
| --- | --- | --- | --- | --- |
| 1 | 400 | 800 | 10 | 4 |
| 2 | 220 | 600 | 20 | 8 |
| 3 | 130 | 500 | 30 | 12 |
| 4 | 85 | 450 | 40 | 16 |
| 5 | 63 | 425 | 50 | 20 |
| 6 | 52 | 413 | 60 | 24 |
| 7 | 46 | 407 | 70 | 28 |
| 8 | 43 | 404 | 80 | 32 |
| Table 1: Numbers of selection candidates and testers used in every step of the selection algorithm. | | | | |

| Iteration | Male selection candidates | Female selection candidates | # female inds in tester trios for males | # male inds in tester trios for males | # female inds in tester trios for females | # male inds in tester trios for females |
| --- | --- | --- | --- | --- | --- | --- |
| 1 | 400 | 800 | 20 | 10 | 4 | 8 |
| 2 | 220 | 600 | 40 | 20 | 8 | 16 |
| 3 | 130 | 500 | 60 | 30 | 12 | 24 |
| 4 | 85 | 450 | 80 | 40 | 16 | 32 |
| 5 | 63 | 425 | 100 | 50 | 20 | 40 |
| 6 | 52 | 413 | 120 | 60 | 24 | 48 |
| 7 | 46 | 407 | 140 | 70 | 28 | 56 |
| 8 | 43 | 404 | 160 | 80 | 32 | 64 |
| Table 2: Numbers of selection candidates and testers used in every step of the selection algorithm when using criterion “ExpBVSelGrGrOff”. | | | | | | |

MAF and AF distribution

|  | Minor allele frequency | Allele frequency |
| --- | --- | --- |
| All variable SNPs with MAF >0.02 | 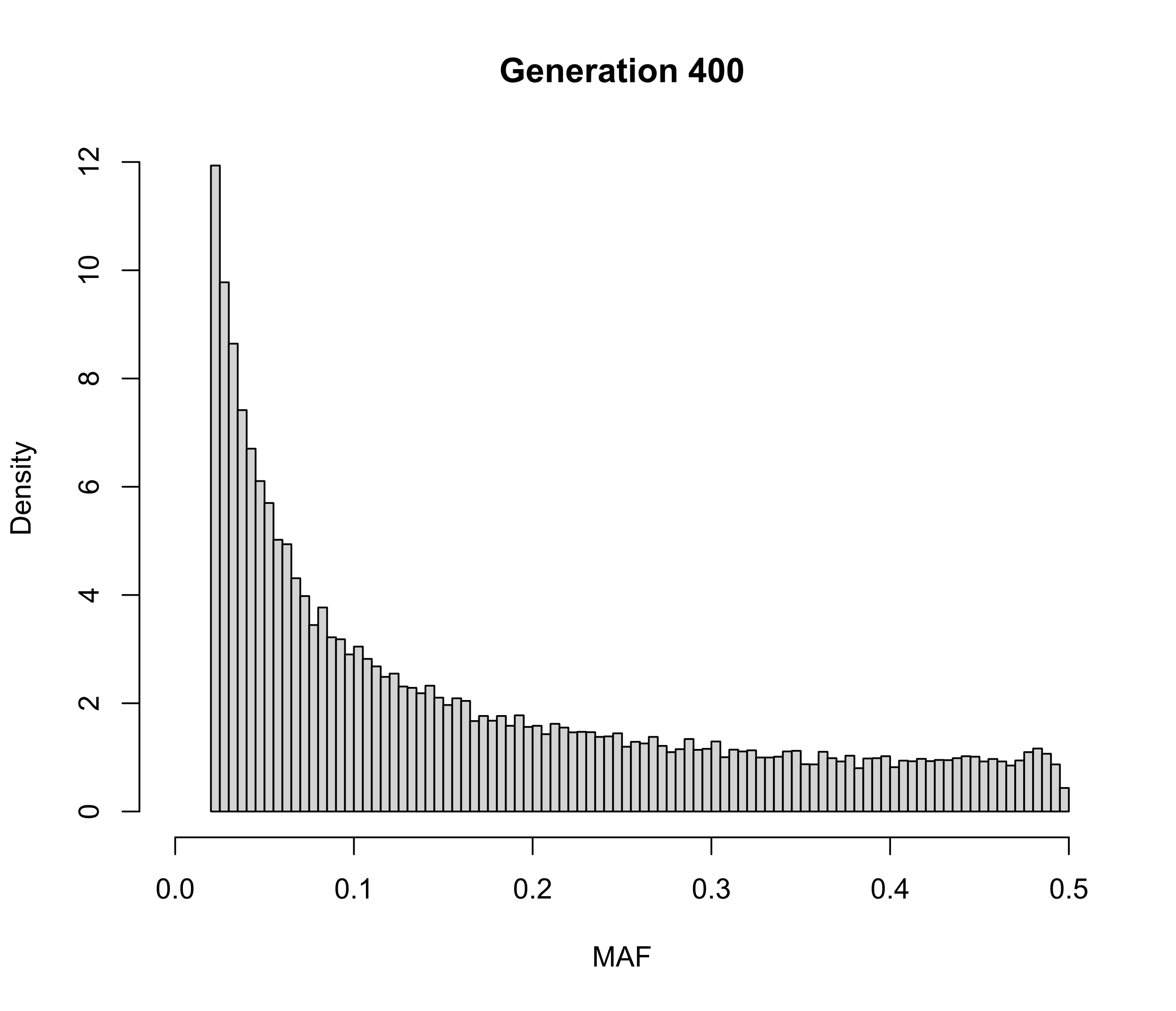  A | 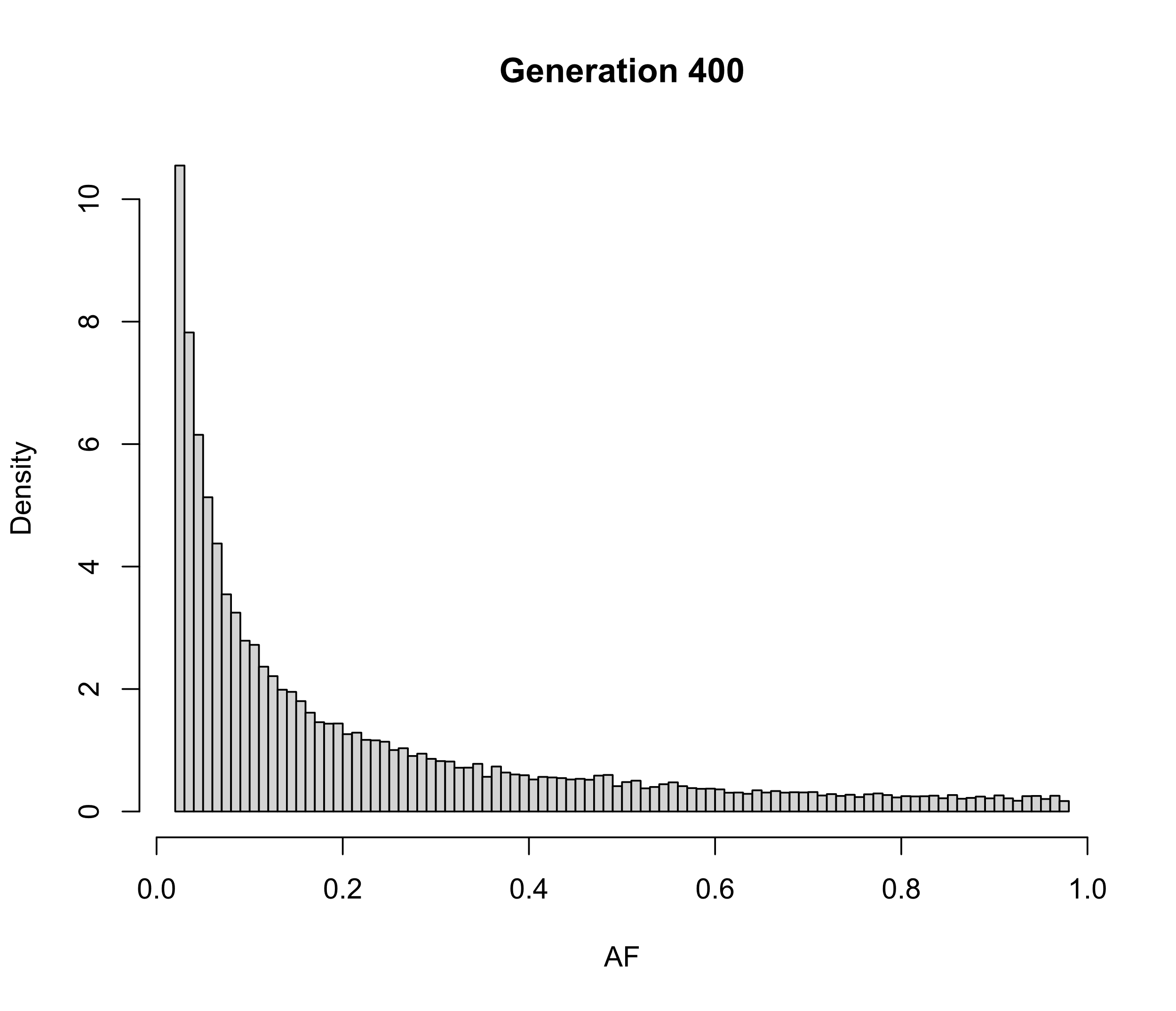  B |
| Selected SNPs for genotyping array | 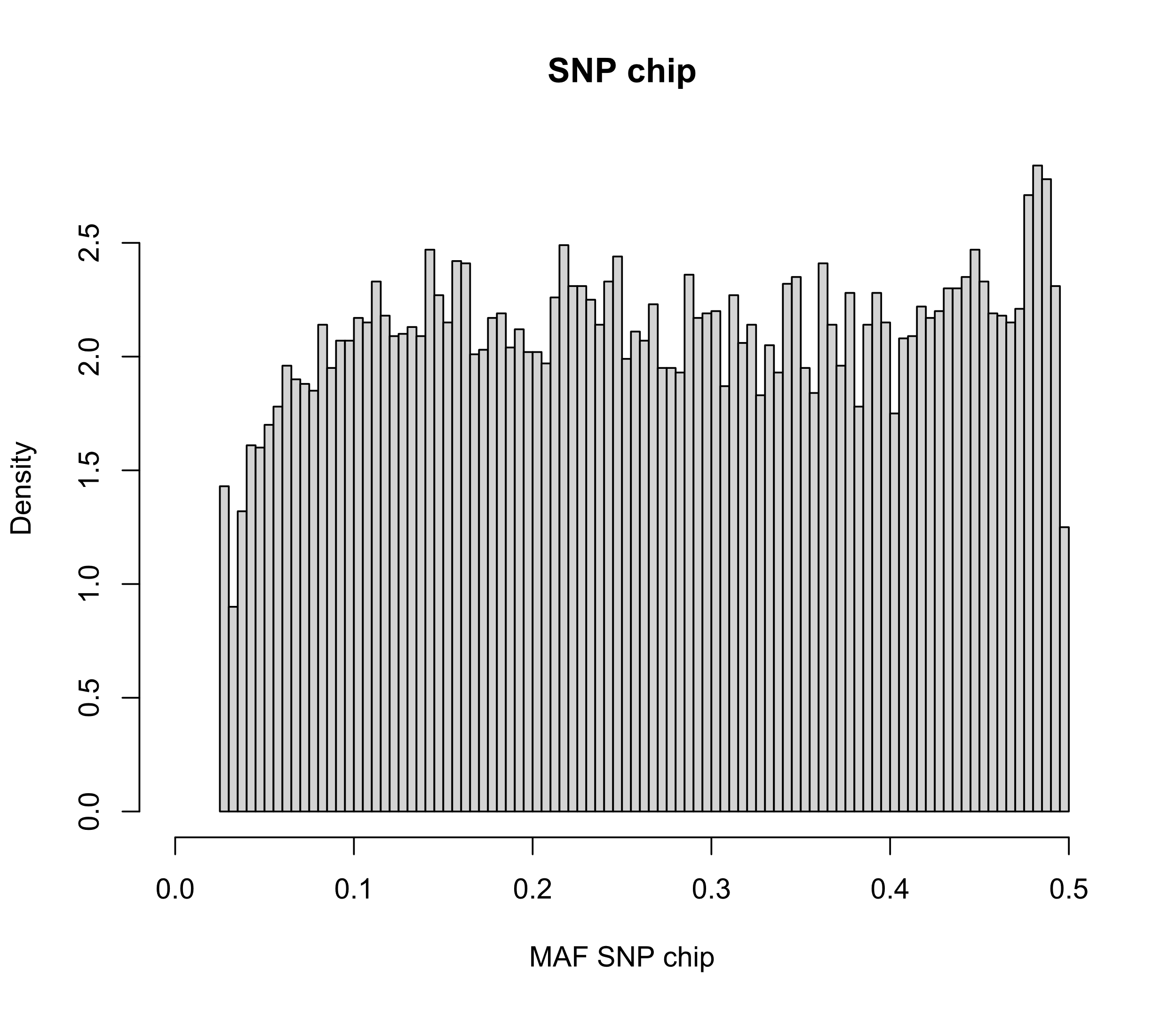  C | 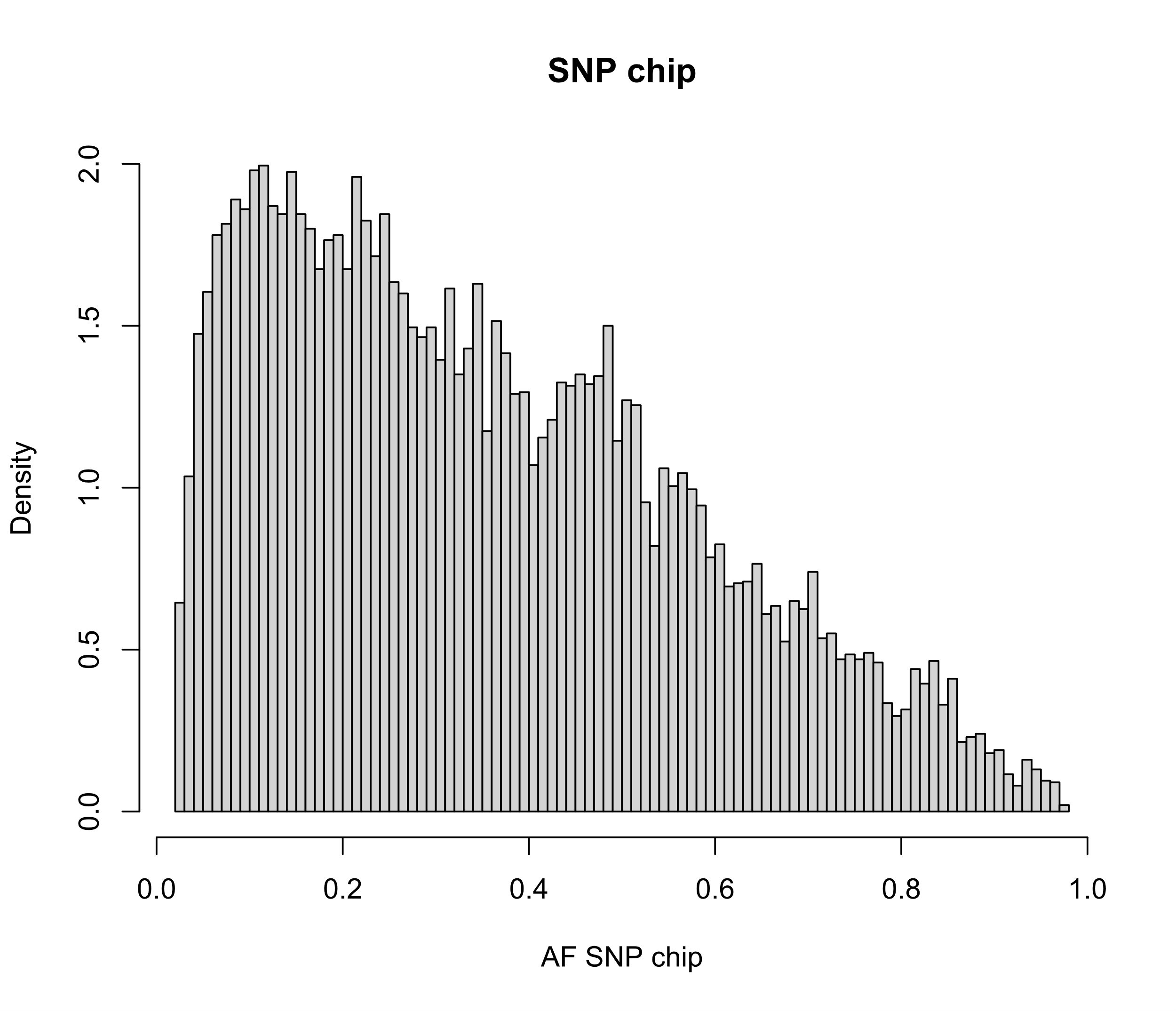  D |
| Supplementary Figure SF1: Distribution of minor allele frequencies (MAF) of all loci (A), distribution of allele frequencies (AF) of all loci (B), distribution of the MAF of 20,000 loci selected for the genotyping array (C) and distribution of the AF of 20,000 loci selected for the genotyping array (D). The AF and MAF refer are observed in the last generation of the population history simulation of a random replicate. Only alleles with a MAF larger than 0.02 were considered. | | |

Genomic prediction accuracies

| Table ST3: Correlation of true with estimated breeding values of selection candidates. | | | | |
| --- | --- | --- | --- | --- |
|  |  | Gen1 | Gen5 | Gen19 |
| 1-generation training population | Random | 0.685 | 0.723 | 0.725 |
|  | GEBV | 0.632 | 0.615 | 0.555 |
|  | Index5 | 0.633 | 0.627 | 0.561 |
|  | ExpBVSelGrOff | 0.635 | 0.627 | 0.570 |
|  | ExpBVSelGrGrOff | 0.639 | 0.633 | 0.580 |
| 3-generation training population | Random | 0.747 | 0.788 | 0.800 |
|  | GEBV | 0.686 | 0.674 | 0.612 |
|  | Index5 | 0.689 | 0.679 | 0.624 |
|  | ExpBVSelGrOff | 0.694 | 0.691 | 0.638 |
|  | ExpBVSelGrGrOff | 0.700 | 0.704 | 0.654 |
| 6-generation training population | Random | 0.772 | 0.821 | 0.833 |
|  | GEBV | 0.714 | 0.705 | 0.651 |
|  | Index5 | 0.720 | 0.714 | 0.674 |
|  | ExpBVSelGrOff | 0.725 | 0.730 | 0.686 |
|  | ExpBVSelGrGrOff | 0.734 | 0.743 | 0.705 |

| Table ST4: Correlation of true with estimated breeding values of all animals in the training population. | | | | |
| --- | --- | --- | --- | --- |
|  |  | Gen1 | Gen5 | Gen19 |
| 1-generation training population | Random | 0.685 | 0.723 | 0.725 |
|  | GEBV | 0.632 | 0.615 | 0.555 |
|  | Index5 | 0.633 | 0.627 | 0.561 |
|  | ExpBVSelGrOff | 0.635 | 0.627 | 0.570 |
|  | ExpBVSelGrGrOff | 0.639 | 0.633 | 0.580 |
| 3-generation training population | Random | 0.789 | 0.791 | 0.815 |
|  | GEBV | 0.850 | 0.844 | 0.800 |
|  | Index5 | 0.849 | 0.844 | 0.803 |
|  | ExpBVSelGrOff | 0.848 | 0.847 | 0.808 |
|  | ExpBVSelGrGrOff | 0.846 | 0.847 | 0.812 |
| 6-generation training population | Random | 0.942 | 0.814 | 0.842 |
|  | GEBV | 0.952 | 0.951 | 0.939 |
|  | Index5 | 0.952 | 0.952 | 0.941 |
|  | ExpBVSelGrOff | 0.952 | 0.951 | 0.942 |
|  | ExpBVSelGrGrOff | 0.952 | 0.951 | 0.941 |

Gametic MSV over genic variance

| Table ST9: Ratio of four times the gametic Mendelian sampling variance over the genic variance. | | | | |
| --- | --- | --- | --- | --- |
|  |  | Gen1 | Gen5 | Gen19 |
| 1-generation training population | Random | 0.944 | 0.952 | 0.966 |
|  | GEBV | 0.935 | 0.897 | 0.793 |
|  | Index5 | 0.935 | 0.905 | 0.807 |
|  | ExpBVSelGrOff | 0.939 | 0.910 | 0.814 |
|  | ExpBVSelGrGrOff | 0.941 | 0.921 | 0.833 |
| 3-generation training population | Random | 0.935 | 0.946 | 0.960 |
|  | GEBV | 0.923 | 0.888 | 0.776 |
|  | Index5 | 0.929 | 0.895 | 0.787 |
|  | ExpBVSelGrOff | 0.938 | 0.919 | 0.811 |
|  | ExpBVSelGrGrOff | 0.943 | 0.935 | 0.837 |
| 6-generation training population | Random | 0.931 | 0.941 | 0.959 |
|  | GEBV | 0.916 | 0.873 | 0.769 |
|  | Index5 | 0.924 | 0.888 | 0.784 |
|  | ExpBVSelGrOff | 0.932 | 0.914 | 0.819 |
|  | ExpBVSelGrGrOff | 0.940 | 0.943 | 0.854 |

Standard errors

| Table ST1: Standard error of the mean IBD homozygosity in selection candidates. | | | | |
| --- | --- | --- | --- | --- |
|  |  | Gen1 | Gen5 | Gen19 |
| 1-generation training population | Random | 0.0017 | 0.0017 | 0.0021 |
|  | GEBV | 0.0019 | 0.0024 | 0.0032 |
|  | Index5 | 0.0019 | 0.0022 | 0.0025 |
|  | ExpBVSelGrOff | 0.0019 | 0.0021 | 0.0024 |
|  | ExpBVSelGrGrOff | 0.0019 | 0.0022 | 0.0027 |
| 3-generation training population | Random | 0.0012 | 0.0012 | 0.0017 |
|  | GEBV | 0.0013 | 0.0018 | 0.0024 |
|  | Index5 | 0.0013 | 0.0017 | 0.0022 |
|  | ExpBVSelGrOff | 0.0013 | 0.0016 | 0.0023 |
|  | ExpBVSelGrGrOff | 0.0013 | 0.0017 | 0.0020 |
| 6-generation training population | Random | 0.0011 | 0.0012 | 0.0016 |
|  | GEBV | 0.0012 | 0.0016 | 0.0019 |
|  | Index5 | 0.0012 | 0.0015 | 0.0020 |
|  | ExpBVSelGrOff | 0.0012 | 0.0015 | 0.0020 |
|  | ExpBVSelGrGrOff | 0.0012 | 0.0015 | 0.0021 |

| Table ST2: Standard error of the mean fraction of animals that are selected based on the criterion that would have also been selected based on GEBV. | | | | | | | |
| --- | --- | --- | --- | --- | --- | --- | --- |
|  |  | Males | | | Females | | |
|  |  | Gen1 | Gen5 | Gen19 | Gen1 | Gen5 | Gen19 |
| 1-generation training population | Random | 0.0024 | 0.0026 | 0.0030 | 0.0018 | 0.0021 | 0.0019 |
|  | GEBV | 0 | 0 | 0 | 0 | 0 | 0 |
|  | Index5 | 0.0040 | 0.0035 | 0.0038 | 0.0007 | 0.0007 | 0.0007 |
|  | ExpBVSelGrOff | 0.0048 | 0.0042 | 0.0048 | 0.0012 | 0.0013 | 0.0013 |
|  | ExpBVSelGrGrOff | 0.0051 | 0.0059 | 0.0060 | 0.0014 | 0.0014 | 0.0017 |
| 3-generation training population | Random | 0.0024 | 0.0025 | 0.003 | 0.0018 | 0.0020 | 0.0019 |
|  | GEBV | 0 | 0 | 0 | 0 | 0 | 0 |
|  | Index5 | 0.0044 | 0.0037 | 0.0038 | 0.0009 | 0.0008 | 0.0008 |
|  | ExpBVSelGrOff | 0.0039 | 0.0050 | 0.0053 | 0.0012 | 0.0012 | 0.0011 |
|  | ExpBVSelGrGrOff | 0.0061 | 0.0055 | 0.0054 | 0.0014 | 0.0016 | 0.0015 |
| 6-generation training population | Random | 0.0026 | 0.0026 | 0.0026 | 0.002 | 0.0020 | 0.0019 |
|  | GEBV | 0 | 0 | 0 | 0 | 0 | 0 |
|  | Index5 | 0.0043 | 0.0042 | 0.0036 | 0.0009 | 0.0009 | 0.0009 |
|  | ExpBVSelGrOff | 0.0057 | 0.0051 | 0.0049 | 0.0012 | 0.0014 | 0.0012 |
|  | ExpBVSelGrGrOff | 0.0058 | 0.0062 | 0.0062 | 0.0015 | 0.0015 | 0.0020 |

| Table ST5: Standard error of the mean average genomic prediction accuracy for selection candidates within full-sib family. | | | | |
| --- | --- | --- | --- | --- |
|  |  | Gen1 | Gen5 | Gen19 |
| 1-generation training population | Random | 0.0036 | 0.0043 | 0.0038 |
|  | GEBV | 0.0041 | 0.0040 | 0.0049 |
|  | Index5 | 0.0038 | 0.0039 | 0.0041 |
|  | ExpBVSelGrOff | 0.0042 | 0.0048 | 0.0044 |
|  | ExpBVSelGrGrOff | 0.0044 | 0.0038 | 0.0044 |
| 3-generation training population | Random | 0.0032 | 0.0032 | 0.0033 |
|  | GEBV | 0.0032 | 0.0033 | 0.0040 |
|  | Index5 | 0.0032 | 0.0032 | 0.0038 |
|  | ExpBVSelGrOff | 0.0028 | 0.0037 | 0.0043 |
|  | ExpBVSelGrGrOff | 0.0031 | 0.0034 | 0.0042 |
| 6-generation training population | Random | 0.0028 | 0.0029 | 0.0030 |
|  | GEBV | 0.0029 | 0.0031 | 0.0036 |
|  | Index5 | 0.0030 | 0.0030 | 0.0036 |
|  | ExpBVSelGrOff | 0.0032 | 0.0027 | 0.0038 |
|  | ExpBVSelGrGrOff | 0.0028 | 0.0030 | 0.0037 |

| Table ST6: Standard error of the average correlation of true with estimated gametic MSV in selection candidates. | | | | |
| --- | --- | --- | --- | --- |
|  |  | Gen1 | Gen5 | Gen19 |
| 1-generation training population | Random | 0.0050 | 0.0056 | 0.0061 |
|  | GEBV | 0.0057 | 0.0054 | 0.0061 |
|  | Index5 | 0.0055 | 0.0061 | 0.0057 |
|  | ExpBVSelGrOff | 0.0062 | 0.0064 | 0.0066 |
|  | ExpBVSelGrGrOff | 0.0061 | 0.0056 | 0.0066 |
| 3-generation training population | Random | 0.0051 | 0.0056 | 0.0055 |
|  | GEBV | 0.0047 | 0.0050 | 0.0058 |
|  | Index5 | 0.0050 | 0.0052 | 0.0060 |
|  | ExpBVSelGrOff | 0.0048 | 0.0056 | 0.0058 |
|  | ExpBVSelGrGrOff | 0.0051 | 0.0050 | 0.0063 |
| 6-generation training population | Random | 0.0046 | 0.0044 | 0.0050 |
|  | GEBV | 0.0048 | 0.0049 | 0.0053 |
|  | Index5 | 0.0046 | 0.0049 | 0.0056 |
|  | ExpBVSelGrOff | 0.0048 | 0.0053 | 0.0060 |
|  | ExpBVSelGrGrOff | 0.0051 | 0.0053 | 0.0066 |

| Table ST8: Standard error of the mean ratio of average gametic MSV over variance expected in full-sib families corrected by inbreeding level. | | | | | | | |
| --- | --- | --- | --- | --- | --- | --- | --- |
|  |  | Ratio estimated effects | | | Ratio true effects | | |
|  |  | Gen1 | Gen5 | Gen19 | Gen1 | Gen5 | Gen19 |
| 1-generation training population | Random | 0.0014 | 0.0013 | 0.0014 | 0.0018 | 0.0017 | 0.0017 |
|  | GEBV | 0.0015 | 0.0015 | 0.0014 | 0.0019 | 0.0018 | 0.0019 |
|  | Index5 | 0.0013 | 0.0013 | 0.0014 | 0.0015 | 0.0016 | 0.0017 |
|  | ExpBVSelGrOff | 0.0014 | 0.0014 | 0.0015 | 0.0018 | 0.0019 | 0.0018 |
|  | ExpBVSelGrGrOff | 0.0014 | 0.0015 | 0.0017 | 0.0020 | 0.0017 | 0.0017 |
| 3-generation training population | Random | 0.0020 | 0.0026 | 0.0021 | 0.0022 | 0.0027 | 0.0022 |
|  | GEBV | 0.0025 | 0.0024 | 0.0021 | 0.0025 | 0.0028 | 0.0024 |
|  | Index5 | 0.0020 | 0.0025 | 0.0024 | 0.0022 | 0.0025 | 0.0024 |
|  | ExpBVSelGrOff | 0.0023 | 0.0021 | 0.0021 | 0.0024 | 0.0024 | 0.0027 |
|  | ExpBVSelGrGrOff | 0.0021 | 0.0020 | 0.0021 | 0.0023 | 0.0026 | 0.0022 |
| 6-generation training population | Random | 0.0022 | 0.0026 | 0.0022 | 0.0023 | 0.0023 | 0.0025 |
|  | GEBV | 0.0023 | 0.0021 | 0.0023 | 0.0026 | 0.0024 | 0.0024 |
|  | Index5 | 0.0025 | 0.0024 | 0.0025 | 0.0025 | 0.0028 | 0.0031 |
|  | ExpBVSelGrOff | 0.0023 | 0.0024 | 0.0024 | 0.0022 | 0.0022 | 0.0029 |
|  | ExpBVSelGrGrOff | 0.0023 | 0.0025 | 0.0021 | 0.0024 | 0.0023 | 0.0021 |

| Table ST7: Standard error of the mean of the average correlation of inbreeding level based on method 1 of VanRaden (2008) with estimated gametic Mendelian sampling variance in selection candidates. | | | | |
| --- | --- | --- | --- | --- |
|  |  | Gen1 | Gen5 | Gen19 |
| 1-generation training population | Random | 0.004 | 0.004 | 0.004 |
|  | GEBV | 0.004 | 0.004 | 0.004 |
|  | Index5 | 0.004 | 0.005 | 0.004 |
|  | ExpBVSelGrOff | 0.004 | 0.004 | 0.005 |
|  | ExpBVSelGrGrOff | 0.005 | 0.004 | 0.004 |
| 3-generation training population | Random | 0.004 | 0.004 | 0.004 |
|  | GEBV | 0.004 | 0.004 | 0.004 |
|  | Index5 | 0.004 | 0.004 | 0.004 |
|  | ExpBVSelGrOff | 0.004 | 0.004 | 0.004 |
|  | ExpBVSelGrGrOff | 0.004 | 0.004 | 0.004 |
| 6-generation training population | Random | 0.004 | 0.004 | 0.004 |
|  | GEBV | 0.004 | 0.004 | 0.004 |
|  | Index5 | 0.004 | 0.004 | 0.005 |
|  | ExpBVSelGrOff | 0.005 | 0.004 | 0.004 |
|  | ExpBVSelGrGrOff | 0.004 | 0.004 | 0.004 |
